# Lessons learned in measuring patient engagement in a Canada-wide childhood disability network

**DOI:** 10.1186/s40900-024-00551-9

**Published:** 2024-02-07

**Authors:** Tatiana Ogourtsova, Miriam Gonzalez, Alix Zerbo, Frank Gavin, Keiko Shikako, Jonathan Weiss, Annette Majnemer

**Affiliations:** 1 Faculty of Medicine and Health Sciences, School of Physical and Occupational Therapy, McGill University, Montreal, QC Canada; 2 Research Center of the Jewish Rehabilitation Hospital, Centre de Santé et de Services Sociaux de Laval, Laval, QC Canada; 3 Centre for Interdisciplinary Research in Rehabilitation of Greater Montreal, Montreal, QC Canada; 4 College of Nursing, Rady Faculty of Health Sciences, University of Manitoba, Winnipeg, MB Canada; 5 Montreal Children’s Hospital, Research Institute of the McGill University Health Center, Montreal, QC Canada; 6 Department of Psychology, York University, Toronto, ON Canada

**Keywords:** Patient participation, Patient-partners, Program evaluation, Research networks, Community-based participatory research

## Abstract

**Background:**

The CHILD-BRIGHT Network, a pan-Canadian childhood disability research Network, is dedicated to patient-oriented research, where numerous stakeholders, including patient-partners, researchers, and clinicians are involved at different levels. The Network is committed to continuously improving the level of engagement and partnerships’ impact. Measuring patient engagement is therefore important in reflecting on our practices and enhancing our approaches. We aimed to measure patient engagement longitudinally and explore in greater depth the perceived benefits, barriers and facilitators, and overall satisfaction with patient engagement, from the perspectives of the different stakeholders.

**Methods:**

Patient engagement was measured using online surveys. In a longitudinal study design over a 3-years period (2018–2020) the Community-Based Participatory Research (CBPR) questionnaire was used. To enrich our understanding of patient engagement in Year 3, we employed the Public and Patient Engagement Evaluation Tool (PPEET) in a cross-sectional, convergent parallel mixed-method study design. Descriptive statistics and a thematic-based approach were used for data analysis.

**Results:**

The CBPR questionnaire was completed by n = 167 (61.4% response rate), n = 92 (30.2% response rate), and n = 62 (14.2% response rate) Network members in Years 1, 2, and 3, respectively. Ninety-five (n = 95, 21.8% response rate) members completed the PPEET in Year 3. CBPR findings demonstrate a stable and high satisfaction level with patient engagement over time, where 94%, 86%, and 94% of stakeholders indicated that the project is a “true partnership” in Years 1, 2, and 3, respectively. In Years 2 and 3, we noted an improvement in patient-partners’ comfort level in sharing their views and perspectives (92% and 91% vs. 74%). An increase in critical reflective trust (i.e., allowing for discussing and resolving mistakes) from Year 1 to 3 was found, both from the perspectives of patient-partners (51–65%) and researchers (48–75%). Using the PPEET, patient engagement factors (i.e., communications and supports for participation, ability to share views and perspectives) and impact were highly rated by most (80–100%) respondents. PPEET’s qualitative responses revealed several patient engagement advantages (e.g., increased projects’ relevance, enhanced knowledge translation), barriers (e.g., group homogeneity), facilitators (e.g., optimal communication strategies), and solutions to further improve patient engagement (e.g., provide clarity on goals).

**Conclusion:**

Our 3-years patient engagement evaluation journey demonstrated a consistent and high level of satisfaction with patient engagement within the Network and identified advantages, barriers, facilitators, and potential solutions. Improvements were observed in members’ comfort in sharing their views and perspectives, along with an increase in critical reflective trust. These findings underscore the Network's commitment to enhancing patient engagement and provide valuable insights for continued improvement and optimization of collaborative efforts.

**Supplementary Information:**

The online version contains supplementary material available at 10.1186/s40900-024-00551-9.

## Abstract (plain language summary)

The CHILD-BRIGHT Network, a Canadian childhood disability research Network, is dedicated to patient-oriented research. It engages more than 300 diverse stakeholders, including patient-partners, researchers, and healthcare professionals. We conducted a 3-years study aimed to measure patient engagement over time and delve into the perceived benefits, barriers, and facilitators from the perspectives of the different members. We administered the Community-Based Participatory Research (CBPR) questionnaire in Years 1–3 (completed by 167, 92, and 62 members, respectively) and the Public and Patient Engagement Evaluation Tool (PPEET) in Year 3 (completed by 95 members). Through the CBPR, we identified in which research processes were Network members involved (e.g., defining the research question, results dissemination), appraised the partnership between researchers and other stakeholders such as patient-partners, and determined the type of trust in this partnership. The use of the PPEET allowed us to explore patient engagement impact and what factors facilitate and limit patient engagement (e.g., communication and supports). CBPR results showed a consistently high satisfaction level with patient engagement, with increased comfort among patient-partners in expressing their views over time, showcasing positive collaborative dynamics. Most stakeholders reported a “true partnership” in their engagement, indicating widespread belief in equitable relationships. Additionally, critical reflective trust, allowing for discussing and resolving mistakes in collaborative working activities, increased over the years, with the highest endorsement in Year 3, demonstrating growing trust among stakeholders. The PPEET findings showed positive ratings for communication, support, and impact of patient engagement. Its qualitative responses identified advantages (e.g., increased project relevance), barriers (e.g., lack of diversity in members’ demographic characteristics), facilitators (e.g., effective communication), and suggested improvements (e.g., ensuring goal clarity). In conclusion, our project showed that the partnership between researchers and patient-partners was beneficial, satisfactory and evolved positively over time. The findings are encouraging provided the breadth of the Network, where hundreds of members are primarily connected virtually. We learned that:

1. It is possible to measure patient engagement in a large Network, both at one point in time and over time, and multiple tools can be used together to get a better picture.
2. Regular evaluations are important to optimize the partnership and its impact.
3. The partnership can be improved and strengthened with time through ongoing collaboration, open communication, and a commitment to address the evolving needs and dynamics of all stakeholders involved.

**Supplementary Information:**

The online version contains supplementary material available at 10.1186/s40900-024-00551-9.

## Background

The CHILD-BRIGHT Network (www.child-bright.ca) is a pan-Canadian patient-oriented research Network that works to create brighter futures for children and youth with brain-based developmental disabilities and their families. Created in 2016, the Network is funded by the Canadian Institutes of Health Research (CIHR) under Canada’s Strategy for Patient-Oriented Research (SPOR) and numerous funding partners across the country. It engages over three hundred members, including researchers and trainees, clinicians, decision-makers, industry partners, youth with disabilities, and caregivers of children/youth with disabilities.

The CHILD-BRIGHT Network is dedicated to patient-oriented research. As Canada’s SPOR endorses the active partnership of patients, researchers, health professionals and decision-makers in research to build a sustainable and accessible health care system that optimizes the health of Canadian citizens, related initiatives require authentic involvement of patients and the public in all phases of investigations [1]. The engagement of patient-partners is recommended to improve the relevance and the overall quality of research, through ensuring that the research team targets issues that are meaningful and important to patients [2, 3]. These invaluable team members can be engaged in all phases of the research process, including but not limited to: developing and prioritizing patient-centred research questions and objectives; guiding the study design and procedures; selecting and adjusting outcome measures and intervention materials; developing parts of the intervention methodologies; advising on optimal recruitment strategies; and guiding as well as actively participating in knowledge mobilization activities [4, 5]. In relation to that, CHILD-BRIGHT’s extensive research program was developed based on research priorities identified by youth with disabilities, caregivers of children/youth with disabilities, and other knowledge users such as frontline clinicians and health care administrators. From 2016 to 2022, CHILD-BRIGHT carried out thirteen multi-centre and SPOR-based projects focusing on three childhood disability themes: (1) Early intervention to promote brain and child development; (2) Strategies to support the mental health of children and families; and (3) Service delivery redesign to address gaps in service.

At CHILD-BRIGHT, we recognize that patients and families are experts on their health experiences and needs; they provide unique expertise on their everyday experiences living with a condition and can share their experiences with the health care system. Various stakeholders including patient-partners (youth with disabilities, parents/caregivers of children/youth with disabilities), researchers and clinicians are involved at different levels of the Network and contribute as committee members and/or as research project team members. Committee members are generally engaged over long-term (i.e., at least 1 year in duration) and contribute to important decision-making within the administration of the Network. For instance, this includes the Citizen Engagement Council, where patient-partners (parents/caregivers, family members, and youth-partners) make up the core of this committee. Its purpose is to offer guidance about ways to initiate, improve and sustain meaningful engagement with the Network’s essential partners and constituencies. Research projects members may have variable engagement (i.e., long- and short-term) and collaborate closely with researchers to ensure their voices and experiences are heard and reflected in the research initiatives.

Patient-oriented research and patient engagement measurement are rapidly evolving fields [6]. Previous work on patient engagement in health care research revealed several perceived benefits and certain challenges of these partnerships from various team members (reviewed in [4]). For example, as partners’ perspectives, preferences, and diverse experiences are considered, it can enrich research pertinence and relevance. Moreover, patient-partners’ insights can enhance participant recruitment and retention, increase trust and engagement between the research team and the community, as well as facilitate and optimize knowledge mobilization. On the other hand, challenges related to power dynamics and communication, balancing partners’ perspectives with scientific rigor, as well as training and education can surface and require proactive management [4]. Overall, balancing these factors is essential to ensure the success and impact of patient-oriented research initiatives. In addition, it highlights the need to evaluate these efforts and their impact to ensure that team members are satisfied with the role and that it aligns with their expectations.

The CHILD-BRIGHT Network is committed to continuously enhancing the partnership with patient-partners throughout the entire research project cycle, spanning from protocol development to findings dissemination. Therefore, measuring the Network’s patient engagement and its impact is an essential ongoing activity. Additionally, findings might inform other teams and/or networks as they plan for or navigate their patient engagement journeys. Our objectives were to: (1) Measure patient engagement longitudinally as the research projects evolved through their cycle (from research protocol and intervention co-development to recruitment, data collection, analysis, manuscript preparation and dissemination); and (2) Explore the perceived benefits, barriers and facilitators and overall satisfaction with patient engagement from the perspectives of the different stakeholder groups, including researchers, committee members, patient-partners, trainees, and youth advisors, among others.

## Methods

### Measuring patient engagement—journey overview

Our patient engagement measurement journey spans over a 3-years period. In 2018 (Year 1), we launched our first evaluation using the Community-Based Participatory Research (CBPR) questionnaire and continued using the CBPR questionnaire in 2019 (Year 2) and 2020’s (Year 3) patient engagement evaluations. In 2020, we aimed to enrich our understanding of patient engagement and proceeded to assess it using the Public and Patient Engagement Evaluation Tool (PPEET). In addition, we conducted individual interviews of researchers and patient-partners [7] and completed a project-specific patient engagement evaluation using the PPEET for one of the CHILD-BRIGHT’s projects [8].

### Engagement of patient-partners in the current project

Patient-partners were engaged in various ways in the current project. One co-author of the present manuscript (FG) is a patient-partner. His role included the conceptualization of the project, selecting measurement tools (including the CBPR questionnaire and the PPEET), providing feedback on the methodology and data collection, validating findings (quantitative and qualitative), guiding and providing feedback on the visualization of findings (specifically, how these were displayed as part of published reports, info briefs, and visual summaries [9–12]), and reviewing and providing valuable feedback on the current manuscript. In addition, in our project-specific patient engagement evaluation [8], patient-partners were involved in modifying the PPEET, and that modified version was used in the current project. More specifically, the following three statements were added by patient-partners in the section of *Communication and supports for participation*:

- “I am satisfied with the compensation I received for my role on the committee.”
- “The committee is generally prepared for meetings (e.g. agenda/questions for the upcoming meeting and minutes of previous meeting are provided ahead to time to review, timelines are outlined, etc.).”
- “I receive information with enough time to read and respond within the context of my work/life schedule.”.

### Study design

For the CBPR questionnaire, we employed a longitudinal survey design with three data collection points in Year 1 (2018), Year 2 (2019), and Year 3 (2020). For the PPEET, we used a cross-sectional, convergent parallel mixed-method study design, including quantitative and qualitative approaches.

### Study population

To ensure a comprehensive assessment, partners within all 13 CHILD-BRIGHT projects, including the parent-advisory group (PAG, caregivers of children/youth with disabilities), youth-advisors (youth/young adults with disabilities), researchers/principal investigators, trainees, committee members, clinicians/health professionals and education professionals (e.g., teachers), consultants, policy makers, and industry partners, from across Canada were invited to participate. This inclusive approach allowed for a thorough patient-engagement evaluation of the SPOR-based initiatives within the context of childhood disability themes. All Network members were eligible to participate, irrespective of their engagement duration. Given the Network's establishment in 2016, the initial patient-engagement evaluation in 2018 primarily involved members who were relatively novice.

### Study procedures

Informed consent was obtained prior to completing the surveys. Approval was granted by the Ethics Committee of the Research Institute of the McGill University Health Center (study no. 2017-2850) and was performed in line with the principles of the Declaration of Helsinki. Participants’ contact information was not connected to their survey responses and their email or Internet protocol addresses were not recorded by the survey system. The survey system assigned a unique identifier to the survey link sent to each participant and recorded only the assigned identifier with each person’s responses. Identifiers were used to connect data but not to connect data to individuals. For all measures, we used an online survey created in the REDCap platform (https://www.project-redcap.org/). Surveys were active for a period of 6 weeks. Two weeks after the initial mail out, a follow-up email reminder was sent to those who had yet to complete the survey. A second email reminder was sent 4 weeks following the initial mail out. These were sent by the CHILD-BRIGHT administration team.

Respondents were asked to indicate the stakeholder group they identified most with (e.g., researcher/principal investigator, PAG, committee member). For the CBPR questionnaire, principal investigators (PIs)/researchers were directed to the *Principal Investigator* version of the CBPR questionnaire, and all other respondents were directed to the *Stakeholder* version. For the PPEET, the participants were directed to one of the four versions of the PPEET, depending on their main stakeholder group (PI, PAG, youth advisor, committee member).

### Measurement of outcomes

#### CBPR

The online survey included the CBPR questionnaire and a section on sociodemographic questions (e.g., education, ethnicity, work status, income). The CBPR is an evaluation tool that examines the degree of stakeholder involvement and partnership in the research process as well as how researchers are engaging stakeholders in their projects [13]. The CBPR questionnaire has been used to assess stakeholder involvement and partnerships across different research areas (e.g., nutrition, substance use, cancer) and with diverse groups of stakeholders (e.g., white, Latino/a, Asian communities) [13]. There is strong factor analytic support for the constructs underlying the CBPR questionnaire [14, 15], and the questionnaire is freely available and used by the research team who developed the tool as well as others conducting CBPR projects.

We used two versions of the CBPR questionnaire: one for PIs (lead researchers involved in CHILD-BRIGHT research projects) and one for stakeholders who interact in different ways with research projects (CHILD-BRIGHT patient-partners or family members, staff or trainees, co-investigators, and committee members). The *Stakeholder* version of the CBPR asked stakeholders about their engagement experiences to assess *partnership development* (involvement in each phase of the project), *partnership appraisal*, and *partnership trust*. By delving into these aspects of partnership, our study aimed to provide a comprehensive understanding of the intricate dynamics between researchers and patient-partners.

- *Partnership development* was assessed across seven involvements, each representing a phase of the research process: (1) Defining the problem; (2) Deciding on which issue to research; (3) Developing research questions; (4) Creating research instruments or guidelines; (5) Collecting data; (6) Analyzing and/or interpreting the data; and (7) Disseminating findings. Stakeholders were asked to rate how involved they have been in each step of the research process using a 5-point Likert scale ranging from “Not at all involved” to “Very involved”. Respondents can also select “Does not apply” for any of the items. To facilitate interpretation, responses were recorded as “No involvement” (Not at all involved or Not very involved) or “Involved” (Somewhat involved, Involved, Very involved).
- For *partnership appraisal*, respondents were asked to rate their agreement across seven items: (1) When the different partners come together, I feel comfortable sharing my opinion; (2) This project is a true partnership; (3) So far, the work of the partnership has been good for the community; (4) So far, the partnership has been good for me personally; (5) I am satisfied with my level of involvement in this project; (6) It is important to me to have my name on presentations and articles; and (7) It is important to have my work acknowledged on presentations and articles. Respondents were asked to rate their level of agreement with each statement using a 5-point Likert scale ranging from “Strongly disagree” to “Strongly agree”. A “Does not apply” response category could be selected for each item. Responses were re-coded to “Disagree” (Strongly disagree, Somewhat disagree) and “Agree” (Somewhat agree, Strongly agree) to create more meaningful groupings for analysis and reporting. Questions about *partnership appraisal* aim to gauge the perceptions and evaluations of respondents regarding the quality and effectiveness of their collaborations. This information is crucial for identifying strengths and areas for improvement in the collaborative processes.
- To assess *trust in the partnership*, respondents were asked to rate the type of trust they have in the partnership using the question: “What type of trust do you think you have now in the partnership?”. Respondents could select from the following response categories: critical reflective (i.e., trust that allows for mistakes and where differences can be talked about and resolved), proxy (i.e., partners are trusted because someone who is trusted invited them), functional (i.e. partners are working together for a specific purpose and timeframe, but mistrust may still be present), neutral (i.e., partners are still getting to know each other; there is neither trust nor mistrust), unearned (i.e., trust is based on member’s title or role with limited or no direct interaction), proxy mistrust (i.e., mistrust in representatives involved in the research process), and no trust. We were interested in the percentage of respondents who noted critical reflective trust, where trust is at the place where mistakes and other issues resulting from differences (in culture; power) can be talked about and resolved. Exploring *partnership trust* is essential as it underlines the importance of a trusting and respectful relationship between researchers and patient-partners. Trust is fundamental for effective collaboration, open communication, and the successful achievement of research goals.

The *PI* version asked researchers about the extent to which they had engaged stakeholders in their project, partnership development (stakeholder involvement in the research process), stakeholder involvement in the partnership, and perceived partnership trust. Instead of asking PIs to reflect on their own involvement, the CBPR questionnaire for PIs asks them to rate how involved stakeholders have been engaged in each step of the research process using the single item with seven response categories described above. The same question used to assess stakeholder trust in the partnership was also used to assess PIs’ trust in the partnership.

### PPEET

Data collection consisted of administering a modified version of a standardized questionnaire, the Public and Patient Engagement Evaluation Tool (PPEET) [16]. This tool has been previously validated, applied in different health care organizations, and more recently refined after a feasibility assessment [17–19]. The PPEET is designed to explore existing enablers and barriers related to patient engagement processes, as well as the impact and influences of patient engagement. The ‘Ongoing/Long Term Engagement Initiative’ section of the PPEET was used in the current study. The questionnaire contains 21 statements or questions for PAG (5/21 are open-ended questions, 3/21 were added as per feedback by our parent-advisors who reviewed the questionnaire before deployment as described above) and 18 questions/statements for the research team in four categories: (1) *Communication and support for participation*; (2) *Sharing views and perspectives*; (3) I*mpacts and influence of the engagement initiative*; and (4) *Final thoughts/satisfaction*. Sixteen statements were rated using a 5-point Likert scale, ranging from ‘strongly disagree’ to ‘Strongly agree’ in both versions used. Each category also includes one to two open-ended questions to further explore respondents’ perspectives (e.g., in *Communication and support for participation*: “*What else would you like us to know about how your participation in was supported*”).

### Data management and analysis

The data was securely stored in REDCap, a password-protected platform, and accessed or reviewed by members of the research team and CHILD-BRIGHT Network staff. To ensure confidentiality, all study participants were anonymized using numerical identifiers, preventing the display or linkage of any identifying information to their profiles, which may contain such details. The CBPR questionnaire’s and PPEET’s quantitative results were explored using univariate descriptive statistics in IBM SPSS Statistics 27. All responses to the open-ended questions of the PPEET were transferred into the NVivo 12 (QRS International, Doncaster, VIC, Australia) software for qualitative analysis. An inductive thematic-based approach [20] was used to analyse the qualitative data as follows: one reviewer (TO) examined all the responses and identified general emerging themes and subthemes. Themes and subthemes were determined based on inductive reasoning, where utterances revealed key descriptors of the phenomenon. The reviewer (TO) then coded each response under the most suitable theme/subtheme (s). Additional subthemes were created or collapsed (in case of overlap in emerging idea) where necessary. The final coding was then verified for accuracy by a second reviewer (MG). Disagreements in categorization of utterances were discussed between the two reviewers; if a decision could not be reached, it was resolved by a third reviewer (AM).

To ensure strong quality in reporting about patient and public involvement, we followed the Guidance for Reporting Involvement of Patient and the Public short form (Additional file 1: S1) [21]. In accordance with these guidelines, the present manuscript provides information on engagement objectives and methods, positive and negative results, impacts and influences of patient engagement, and the team’s critical perspective on the experience.

## Results

Table 1 displays the distribution of stakeholder groups per year. The Network included n = 272, 305, and 436 members in Years 1, 2, and 3, respectively, demonstrating consistent Network growth over time. This expansion was primarily driven by a rise in patient-partners, including youth advisors (+ 51 new members between Years 1 and 3), researchers (+ 12), research staff (+ 38), trainees (+ 33), and committee members (+ 10).

**Table 1:** Stakeholder groups per year

Stakeholder group	Year 1 2018	Year 2 2019	Year 3 2020
	n (%)	n (%)	n (%)
Researchers/investigators	99 (36.4)	101 (33.1)	111 (25.5)
Research staff	42 (15.4)	48 (15.7)	80 (18.3)
Committee members	44 (16.2)	41 (13.4)	54 (12.4)
Patient-partners (parents/caregivers)	44 (16.2)	60 (19.7)	87 (20.0)
Youth advisors	2 (0.7)	2 (0.7)	10 (2.3)
Trainees	16 (5.9)	26 (8.5)	49 (11.2)
Education/health professionals	9 (3.3)	8 (2.6)	20 (4.6)
Support services/consultants	14 (5.1)	15 (4.9)	14 (3.2)
Policy makers	2 (0.7)	4 (1.3)	8 (1.8)
Industry partners	–	–	3 (0.7)
Total n (%)	272 (100)	305 (100)	436 (100)

The numbers indicate the primary stakeholder group to which each member belongs. For instance, a policy maker (primarily) who also serves as a researcher would not be included in the Total count for “Researchers.” The numbers for committee members exclude patient-partners and youth-advisors

### CBPR

#### Survey respondents

The Total number of respondents who completed the CBPR by stakeholder group per year is outlined in Table 2. Response rates were 61.4%, 30.2%, and 14.2% for Years 1, 2, and 3, respectively. In Year 1 (2018), many respondents still were unsure of their role in the Network, thus selected ‘Not specified’. While we show the number of PIs as a stakeholder group, we report on their results separately from other stakeholders, given their different position within the CHILD-BRIGHT Network and their use of the PI version of the CBPR tool.

**Table 2:** CBPR survey respondents by stakeholder group and year

Stakeholder group	Year 1 2018^a^	Year 2 2019^b^	Year 3 2020^c^
	n (%)	n (%)	n (%)
Collaborating investigator	17 (10)	19 (20.7)	13 (21.0)
Committee members	5 (3)	7 (7.6)	11 (17.7)
Patient partner	11 (7)	21 (22.8)	10 (16.1)
Staff/trainer	17 (10)	15 (16.3)	6 (9.7)
Project team member	28 (17)	15 (16.3)	7 (11.3)
Principal investigators	23 (14)	11 (12.0)	12 (19.4)
Other	5 (3)	1 (1.1)	2 (3.2)
Not specified	61 (37)	3 (3.3)	1 (1.6)
Total n (%)	167 (100.0)	92 (100.0)	62 (100.0)

#### Stakeholder involvement and appraisal of the partnership

Table 3 presents the percentage of stakeholders (other than PIs) who rated the CBPR items of interest at each of the three data time points. In Year 1, the most common involvements in research process occurred in areas of collecting data (65%) and creating instruments or guidelines (55%). The highest level of positive appraisal of the partnership was reflected by respondents “*[being] satisfied with [the] level of involvement in this project*” (83%). In Year 2, the most common involvements in research process were in areas of in collecting data (63%), followed by creating research instruments or guidelines (57%), while the highest level of positive appraisal of the partnership was reflected by respondents “*feeling comfortable sharing [their] opinion when the different partners come together*” (91%); being “*satisfied with [the] level of involvement in this project*” (83%); and reporting that “*so far, the work of the partnership has been good for the community*” (83%). In 2020, the most common involvements in research process were in the areas of creating research instruments or guidelines (66%) and disseminating findings (64%). Similarly, the highest level of positive appraisal of the partnership was reflected by respondents “*feeling comfortable sharing [their] opinion when the different partners come together*” (92%); being “*satisfied with [the] level of involvement in this project*” (81%); and reporting that “*so far, the work of the partnership has been good for the community*” (85%). Over the years, most stakeholders (94, 86, 94% in Year 1, 2, and 3 respectively) reported felt that the project was a “*true partnership*” (i.e., collaborative, and equitable relationship between stakeholders) while endorsements of critical reflective trust (i.e., trust that allows for mistakes and where differences can be talked about and resolved) was highest in Year 3.

**Table 3:** Stakeholder involvement and appraisal of the partnership

CBPR item of interest	Year 1 2018 N = 83	Year 2 2019 N = 78	Year 3 2020 N = 48
*Involvement in the research process*^*a*^			
Defining the problem	49	51	56
Deciding on which issue to research	33	45	52
Developing research questions	41	53	58
Creating research instruments or guidelines	55	57	66
Collecting data	65	63	63
Analyzing and/or interpreting the data	36	51	48
Disseminating and sharing findings	40	53	64
*Appraisal of the Partnership*^*b*^			
When the different partners come together, I feel comfortable sharing my opinion	74	91	92
This project is not a true partnership	6	14	6
So far, the work of the partnership has been good for the community	68	83	85
So far the partnership has been good to me personally	67	78	77
I am satisfied with my level of involvement in this project	83	83	81
It is important to me to have my name on presentations and articles	48	60	50
It is important to me to have my work acknowledged on presentations and articles	48	57	51
*What type of trust do you think you have now in this partnership?*^c^	51	47	65

The data reported in this table represent different samples per year

^a^Percentage of respondents who answered: Somewhat Involved/Involved/Very involved

^b^Percentage of respondents who answered: Somewhat/Strongly agree

^c^Percentage noting critical reflective trust. Responses are for stakeholders who indicated one of the stakeholder types that were not research/research teams and who indicated another stakeholder type

#### PIs’ appraisal of stakeholder involvement and partnership trust

Table 4 summarizes how PIs rated the CBPR items of interest per year. Over the 3 years, 92–100% PIs reported that patient-partners’ involvements in research process were in the areas of defining the problem, deciding on which issue to research, and developing research questions. Also, in Year 2 and 3, PIs reported common patient-partner involvements in the areas of collecting data and creating research instruments or guidelines. Appraisals of the partnership were also positive across items (ranging from 70 to 100%), while the endorsement of the highest level of trust was uppermost in Year 3 (75%).

**Table 4:** Researchers/principal investigator appraisal of stakeholder involvement and partnership trust

CBPR Items	Year 1 2018 N = 23	Year 2 2019 N = 11	Year 3 2020 N = 12
*Involvement in the research Process*^*a*^			
Defining the problem	100	100	92
Deciding on which issue to research	96	100	92
Developing research questions	96	100	92
Creating research instruments or guidelines	82	91	92
Collecting data	67	100	83
Analyzing and/or interpreting the data	38	50	50
Disseminating and sharing findings	33	44	75
*Appraisal of the Partnership*^*b*^			
When the different partners come together, I feel that stakeholders are comfortable in sharing their opinion	96	100	100
This project is not a true partnership	9	10	0
So far, the work of the partnership has been good for the community^b^	78	91	75
So far the partnership has been good to me personally	91	91	92
I am satisfied with my level of supervision in this project^b^	91	73	100
It is important to me to have stakeholders’ names on presentations and articles	70	91	83
It is important to me to have stakeholders’ work acknowledged on presentations and articles	96	100	92
*What type of trust do you think you have now in this partnership?*^*c*^	48	36	75

The data reported in this table represent different samples per year

^a^Percentage of respondents who answered: Somewhat Involved/Involved/Very involved

^b^Percentage to respondents who answered: Somewhat/Strongly agree

^c^Percentage of respondents who indicated having critical reflective trust in the partnership

### PPEET

#### Survey respondents

Table 5 outlines the PPEET survey respondents’ stakeholder group distribution and their engagement duration. While Network 106 members (n = 31 committee members, n = 9 patient-partners on committees, n = 18 patient-partners on projects, n = 44 researchers, and n = 4 youth advisors) started the survey, the responses of 95 participants (21.8% response rate) were recorded and considered in the analysis. They included researchers (n = 44 across 12 research projects), committee members (n = 31, across 6 committees), patient-partners on projects (n = 18/61, across 8 projects), patient-partners on committees (n = 8/26, across 4 committees), and youth advisors (n = 4). The response rate per stakeholder group was modest at 39.6% for researchers, 29.5% for patient-partners on research projects, 30.7% for patient-partners on committees, 57.4% for committee members, and 40% for youth advisors. We noted that 67.3% of respondents were members of their respective stakeholder group since the beginning of the project, whereas 23.2% were novice members who have joined the teams within the year preceding recruitment. Exceptionally, all youth advisors were novice members. Researchers included, but were not limited to, principal investigators (n = 10, 23.2%), collaborating investigators (n = 11, 25.5) and research assistants (n = 11, 25.5%). Most patient-partners on projects were parents of children/youth with disability (n = 13, 76.4%).

**Table 5:** PPEET survey respondents by stakeholder group and their project engagement duration

Stakeholder Group		Number of respondents n (%)	Project engagement duration		
			Since the beginning of the project n (%)	Within the last year n (%)	Other n (%)
Researchers	Principal investigator: 10 (23.2)	43 (45.2)	31 (70.5)	7 (15.9)	5 (11.6)
	Collaborating investigator: 11 (25.5)				
	Research assistant: 11 (25.5)				
	Trainee: 6 (13.69)				
	Project coordinator: 3 (6.9)				
	Other: 2 (4.6)				
Committee members	Committee member: 20 (80.0)	25 (26.3)	17 (54.8)	6 (19.4)	2 (7.6)
	Chair/Vice-chair: 4 (16.0)				
	Other (e.g. non-voting member: 1 (4.0)				
Patient partners on projects	Parent: 13 (76.4)	17 (17.8)	13 (72.2)	3 (16.7)	1 (5.8)
	Youth: 3 (17.6)				
	Other: 1 (5.8)				
Patient partners on committee	Parent: 3 (50.0)	6 (6.3)	3 (33.3)	2 (22.2)	1 (16.6)
	Youth: 2 (30.0)				
	Other: 1 (20.0)				
National youth advisory panel		4 (4.2)	0 (0)	4 (4)	0 (0)
TOTAL		95 (100)	64 (67.3)	22 (23.2)	9 (9.4)

#### Quantitative outcomes

**Fig. 1:**
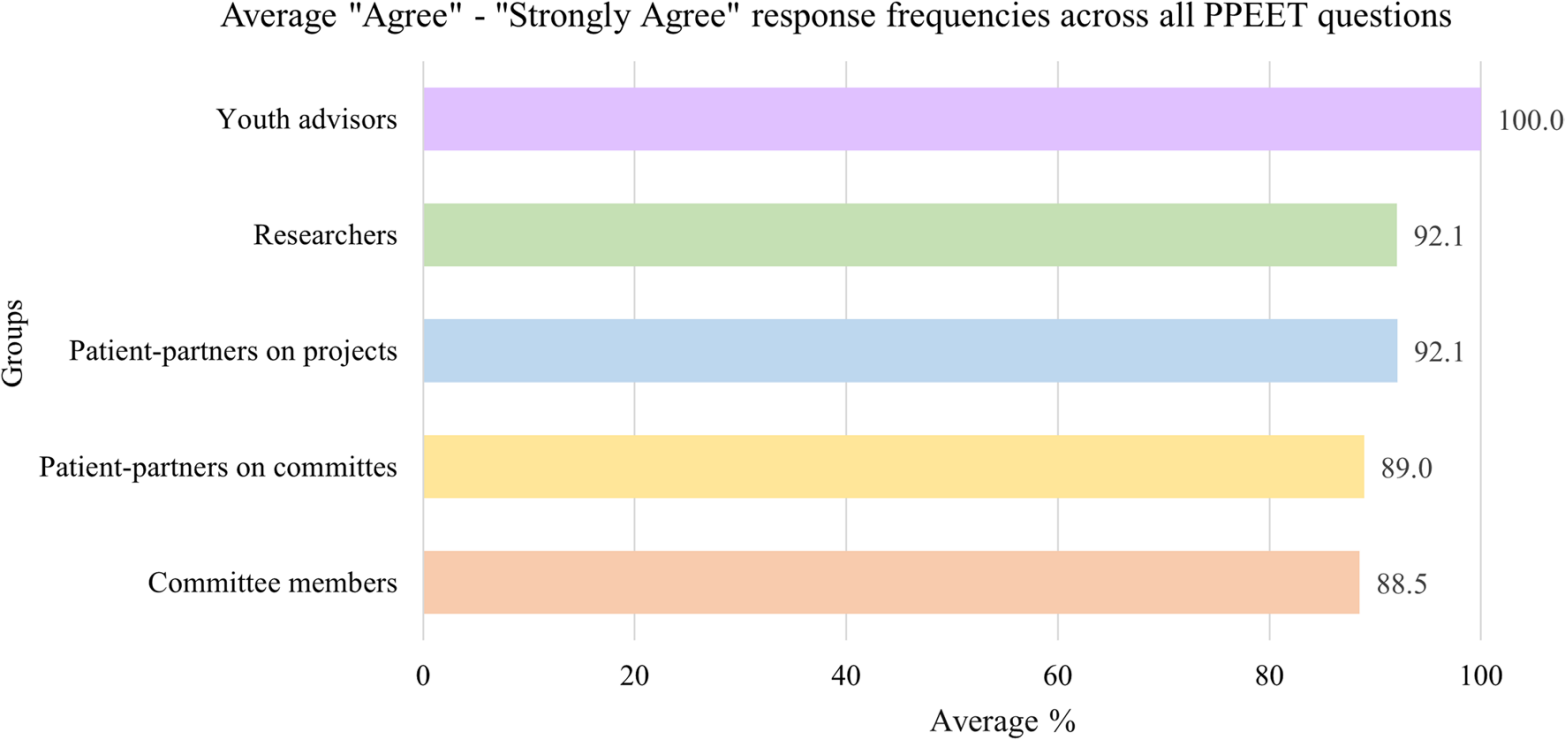
Average “Agree”—“Strongly Agree” response frequencies across all PPEET questions

Figure 1 illustrates the average response frequencies for different group members. We note that most stakeholders (88.5–100% of survey respondents) ‘agreed’ to ‘strongly agreed’ with patient engagement elements assessed through the PPEET, suggesting positive and strong patient engagement processes and impact in the Network. The average ‘agreed’ to ‘strongly agreed’ response rate across all PPEET questions for all respondent groups combined is high at 92.7%.

Additional file 1: S2 provides detailed response frequencies to all PPEET questions for the four different stakeholder groups. Patient-partners on committees generally ‘agreed—strongly agreed’ (83.3–100%) with all PPEET items related to *communication and supports for participation*, except for their “*[satisfaction] with the compensation [they] receive for [their] role on the committee*”, where 66.7% ‘agreed-strongly agreed’. In *sharing views & perspectives*, 100% of patient-partners on committees ‘agreed-strong agreed’ that they were “*able to express their views freely*” and reported feeling that “*their views are heard*”, while 83.3% ‘agreed—strongly agreed’ that “*a wide range of views are shared on the topics discussed during meetings/activities*”, and that they have “*[brought] forward a broad range of perspectives of the discussion topics*”. In *impacts & influences of engagement initiative*, 80–100% of patient-partners ‘agreed-strongly agreed’ that the “*committee has achieved or is on the right path to achieve its objectives*”, that they are “*confident that [their] input provided as a patient-partners will be used by the committee*”, and that “*the input provided will make a difference in the work of the committee*”. Lastly, in *final thoughts*,100% of patient-partners on committees reported that they are better informed about SPOR, while 80% are “*satisfied with their role*” and report that their “*role as a patient-partner on the committee is a good use of [their] time*”. For patient-partners on projects (n = 18), PPEET results were very similar to those on committees, ranging from 80 to 100% of participants indicating an agreement. However, one patient-partner reported that they ‘strongly disagree’ with being “*able to express their views clearly*”, and one respondent “disagreed” that “*the individuals participating as patient-partners in the project bring forward a board range of perspectives on the discussion topics*”. Youth advisors (n = 4, 100%) agreed with all the PPEET items. Similarly, 85–100% of researchers and 83.3–95.8% of committee members ‘agreed—strongly agreed’ with all the PPEET items.

#### Qualitative outcomes

Tables 6, 7, 8 and 9 summarize the emergent themes/subthemes. Examples of most salient quotes for each theme and subtheme are available in Additional file 1: S3. Four main themes were identified: (1) Advantages of patient engagement (PE), (2) Barriers to PE (3) Facilitators to PE and (4) Strategies to improve PE. Two-hundred-and-forty-eight (n = 248) utterances were analysed, where 44% were related to advantages of PE, 21.8% and 16.1% were barriers and facilitators respectively, and 18.1% were improvement strategies. Amongst the groups, researchers provided the most responses with 57.7% of the utterances, followed by the committee members (32.3%), and patient-partners (10%).

**Table 6:** PPEET results: Advantages of patient engagement (PE)

Theme n = number of utterances	Subtheme n = number of utterances % of Theme	Sub-subtheme n = number of utterances % of Sub-subtheme	Description	PAG utterances n (%)	RES utterances n (%)	COMM utterancesn (%)	Total utterances n (%)
ADVANTAGES OF PE N = 109	Connections N = 4 (3.7)	Collaboration opportunities and human connections N = 2 (50.0)	Refers to not only collaborating with others but also enjoying the overall collaboration experience	2	0	0	2
			Total	2 (100.0)	0 (0.0)	0 (0.0)	(100.0)
		Close communication with research team N = 1 (25.0)	Refers to being able to get first-hand experience with research and better understanding it	1	0	0	1
			Total	1 (100.0)	0 (0.0)	0 (0.0)	1 (100.0)
		Bridging gaps between people involved N = 1 (25.0)	Refers to ensuring that everyone involved is aware of what is going on	0	0	1	1
			Total	0 (0.0)	0 (0.0)	1 (100.0)	1 (100.0)
	Learning and voicing N = 12 (11.0)	For PP—having a chance to advocate for self and peers N = 3 (25.0)	Refers to developing knowledge about how to advocate and then being able to implement these newly learned strategies	0	0	3	3
			Total	0 (0.0)	0 (0.0)	3 (100.0)	3 (100.0)
		Learning about others’ perspectives and CEC mandate N = 2 (16.7)	Refers to the general sentiment about appreciating learning from others	0	1	1	2
			Total	0 (0.0)	1 (50.0)	1 (50.0)	2 (100.0)
		Learning about SPOR N = 6 (50.0)	Refers to learning about SPOR from all perspectives and how to best implement it	0	2	4	6
			Total	0 (0.0)	2 (33.3)	4 (66.7)	6 (100.0)
		Quick adaptation to the changes in interactions related to the pandemic N = 1 (8.3)	Refers to members working hard to ensure that interactions were maintained despite the pandemic	0	1	0	1
			Total	0 (0.0)	1 (100.0)	0 (0.0)	1 (100.0)
	Research project benefits N = 93 (85.3)	Improved recruitment and or research methodology N = 14 (15.1)	Refers to the improvements that have been made to various research projects when considering the PAG’s input and perspective	0	14	0	14
			Total	0 (0.0)	14 (100.0)	0 (0.0)	14 (100.0)
		Improved relevance of knowledge translation and dissemination N = 15 (16.1)	Refers to the ways PAGs and COMs have greatly improved knowledge translation and communication within their respective research projects	1	11	3	15
			Total	1 (6.7)	11 (73.3)	3 (20.0)	15 (100.0)
		Keeping the research team on track N = 1 (1.1)	Refers to COM ensuring that the research team is following a respected timeline and knows the extent of what research is expected to be done	0	0	1	1
			Total	0 (0.0)	0 (0.0)	1 (100.0)	1 (100.0)
		Pilot testing and modifications to the protocol N = 2 (2.2)	Refers to the important role played by participants in the implementation of the research process	0	2	0	2
			Total	0 (0.0)	2 (100.0)	0 (0.0)	2 (100.0)
		PP providing input on how to better engage patients N = 1 (1.1)	Refers to helping implement methods so that patients can all be engaged in the projects in some way or another	0	0	1	1
			Total	0 (0.0)	0 (0.0)	1 (100.0)	1 (100.0)
		PP steering actions right from the start N = 1 (1.1)	Refers to ensuring patients are at the forefront of research projects right away	0	0	1	1
			Total	(0.0)	0 (0.0)	1 (100.0)	1 (100.0)
		Understanding gaps in knowledge that needs to be filled N = 2 (2.2)	Refers to PAGs being able to identify gaps in the knowledge and advise research team of these gaps early	0	0	2	2
			Total	0 (0.0)	0 (0.0)	2 (100.0)	2 (100.0)
		Valuable input making research more relevant, and representative of end users’ needs N = 57 (61.3)	Refers to the time and feedback that has come from PAGs and COMs which have led to positive research developments and implementation of more user-friendly strategies	4	40	13	57
			Total	4 (7.0)	40 (70.2)	13 (22.8)	57 (100.0)
			Total	8 (7.3)	71 (65.1)	30 (27.5)	109 (100)

*PAG* Patient-advisory group, *RES* researchers, *COMM* committee members, *PP* patient-partners

**Table 7:** PPEET results: Barriers to patient engagement (PE)

Theme n = number of utterances	Subtheme n = number of utterances % of Theme	Sub-subtheme n = number of utterances % of Sub-subtheme	Description	PAG utterances n (%)	RES utterances n (%)	COMM utterances n (%)	Total utterances n (%)
Barriers to PE N = 54	Ambiguity in goals N = 1 (1.9)		Refers to ambiguous goals and how to potentially overcome	0	0	1	1
			Total	0 (0.0)	0 (0.0)	1 (100.0)	1 (100.0)
	Availabilities of PP N = 2 (3.7)		Refers to being more transparent from the beginning for planning the expected time and type of engagement	0	2	0	2
			Total	0 (0.0)	2 (100.0)	0 (0.0)	2 (100.0)
	Compensation issues N = 1 (1.9)		Refers to an administrative issue that came up when reimbursing a PAG	0	1	0	1
			Total	0 (0.0)	1 (100.0)	0 (0.0)	1 (100.0)
	Complexity of protocols and research steps N = 2 (3.7)		Refers to lack of transparency surrounding the type of knowledge needed to be a PAG (expectations)	0	1	1	2
			Total	0 (0.0)	1 (50.0)	1 (50.0)	2 (100.0)
	Concurrent commitments impending engagement N = 2 (3.7)		Refers to not being able to reach all voices perhaps due to too many commitments	0	2	0	2
			Total	0 (0.0)	2 (100.0)	0 (0.0)	2 (100.0)
	Deciding what perspectives from PP are to be incorporated and how N = 4 (7.4)		Refers to the challenges of knowing what to incorporate and touches upon methods taken to alleviate (ex: discussions, outlines and documenting everything)	0	3	1	4
			Total	0 (0.0)	3 (75.0)	1 (25.0)	4 (100.0)
	Difficulty understanding the science involved and the research process N = 1 (1.9)		Refers to challenges with understanding concretely goals and timelines, behind the science discussions	1	0	0	1
			Total	1 (100.0)	0 (0.0)	0 (0.0)	1 (100.0)
	Engagement as challenge N = 2 (3.7)		Refers to challenges engaging everyone at the same time	0	0	2	2
			Total	0 (0.0)	0 (0.0)	2 (100.0)	2 (100.0)
	Group homogeneity N = 8 (14.8)		Refers to similar voices being heard, all participants mostly being parents, lack of minority voices	0	5	3	8
			Total	0 (0.0)	5 (62.5)	3 (37.5)	8 (100.0)
	Keep up with engagement over long periods of time N = 2 (3.7)		Refers to engagement dropping off as the study progresses and scheduling difficulties	0	2	0	2
			Total	0 (0.0)	2 (100.0)	0 (0.0)	2 (100.0)
	Lack of clarity re roles N = 5 (9.3)		Refers to PAG’s not knowing what their research is being useful for, or just not understanding their role/context	1	0	4	5
			Total	1 (20.0)	0 (0.0)	4 (80.0)	5 (100.0)
	Lack of F2F meetings N = 1 (1.9)		Refers to not meeting in person	1	0	0	1
			Total	1 (100.0)	0 (0.0)	0 (0.0)	1 (100.0)
	Lack of meetings or PP presence in meetings N = 3 (5.6)		Refers to not seeing PP during some meetings, but also brings up lack of meetings in general, so no opportunity to see PP	0	0	3	3
			Total	0 (0.0)	0 (0.0)	3 (100.0)	3 (100.0)
	Lack of PI engagement N = 1 (1.9)		Refers to not seeing engagement from the core research group during meetings	1	0	0	1
			Total	1 (100.0)	0 (0.0)	0 (0.0)	1 (100.0)
	Linguistic, social and cultural differences N = 1 (1.9)		Refers to the reasons why some PPs may not participate in the meetings to their full extent	0	1	0	1
			Total	0 (0.0)	1 (100.0)	0 (0.0)	1 (100.0)
	Managing PP expectations vs. feasibility in incorporating feedback N = 2 (3.7)		Refers to the challenges of incorporating feedback and also setting limits re how much can efficiently be incorporated	0	2	0	2
			Total	0 (0.0)	2 (100.0)	0 (0.0)	2 (100.0)
	Newsletters hard to navigate N = 1 (1.9)		Refers to challenges with newsletters	1	0	0	1
			Total	1 (100.0)	0 (0.0)	0 (0.0)	1 (100.0)
	Pandemic related issues N = 1 (1.9)		Refers to being more sensitive with regard to reaching out the PPs during pandemic vs how you would reach out in the past	0	1	0	1
			Total	0 (0.0)	1 (100.0)	0 (0.0)	1 (100.0)
	PE is time consuming N = 1 (1.9)		Refers to the length of time it takes to increase PP engagement	0	1	0	1
			Total	0 (0.0)	1 (100.0)	0 (0.0)	1 (100.0)
	PIs too focused on individual research projects N = 1 (1.9)		Refers to lack of engagement from PI/research team and how it can negatively affect PE/effective PE	0	0	1	1
			Total	0 (0.0)	0 (0.0)	1 (100.0)	1 (100.0)
	Planning ahead for resources needed to do engagement N = 1 (1.9)		Refers to the expectation that PE can be done immediately, but really needs investing time and energy to do well	0	1	0	1
			Total	0 (0.0)	1 (100.0)	0 (0.0)	1 (100.0)
	Possibly overwhelming PP with tasks N = 1 (1.9)		Refers to how PAGs participation is currently solicited and how to change that for the future	0	1	0	1
			Total	0 (0.0)	1 (100.0)	0 (0.0)	1 (100.0)
	Project related technical difficulties N = 1 (1.9)		Refers to gaps in communication with PAGs when things go wrong or get delayed with research	0	1	0	1
			Total	0 (0.0)	1 (100.0)	0 (0.0)	1 (100.0)
	Scheduling and organizing meetings that suit everyone’s availabilities N = 4 (7.4)		Refers to the overall difficulties that have arisen with scheduling and solutions like rotation lunch times or offering multiple locations/times	0	4	0	4
			Total	0 (0.0)	4 (100.0)	0 (0.0)	4 (100.0)
	Struggle to establish formal relationships with PP N = 1 (1.9)		Refers to relationships with PP and RES mostly informal, less engagement when reaching out formally	0	1	0	1
			Total	0 (0.0)	1 (100.0)	0 (0.0)	1 (100.0)
	Time zones and different locations N = 2 (3.7)		Refers to logistical challenges when meetings include PAGs from across Canada	1	1	0	2
			Total	1 (50.0)	1 (50.0)	0 (0.0)	2 (100.0)
	Transition in project phases and its impacts on engagement N = 2 (3.7)		Refers to potential measures to take as a way of better planning for changes in engagement levels throughout the project	0	2	0	2
				0 (0.0)	2 (100.0)	0 (0.0)	2 (100.0)
			Grand total	6 (11.1)	32 (59.3)	16 (29.6)	54 (100.0)

*PAG* Patient-advisory group, *RES* researchers, *COMM* committee members, *PP* patient-partners

**Table 8:** PPEET results: Facilitators to patient engagement (PE)

Theme n = number of utterances	Subtheme n = number of utterances % of Theme	Sub-subtheme n = number of utterances % of Sub-subtheme	Description	PAG utterances n (%)	RES utterances n (%)	COMM utterances n (%)	Total utterances n (%)
Facilitators N = 40	Communication strategies N = 15 (37.5)	Adequate preparation for meetings N = 11 (73.3)	Refers to the preparation put in advance from COMM members to ensure meetings are conducted smoothly and allowing all PAGs and COMMs to be heard if they want to comment	0	5	6	11
			Total	0 (0.0)	5 (45.5)	6 (54.5)	11 (100.0)
		Consistent and clear communication N = 1 (6.7)	Refers to the quality of information being shared	1	0	0	1
			Total	1 (100.0)	0 (0.0)	0 (0.0)	1 (100.0)
		F2F meetings N = 1 (6.7)	Refers to the benefits the team felt by having F2F meetings	1	0	0	1
			Total	1 (100.0)	0 (0.0)	0 (0.0)	1 (100.0)
		Pauses in meetings for questions, encouragement for questions N = 1 (6.7)	Refers to how meetings are conducted, and how members feel encouraged to ask questions	0	0	1	1
			Total	0 (0.0)	0 (0.0)	1 (100.0)	1 (100.0)
		Smaller group meetings for better collaborations N = 1 (6.7)	Refers to where the best collaboration was seen	1	0	0	1
			Total	1 (100.0)	0 (0.0)	0 (0.0)	1 (100.0)
	Improved supports to PP N = 2 (5.0)		Refers to how Child Bright has made changes to ensure PP’s are supported to participate	0	2	0	2
			Total	0 (0.0)	2 (100.0)	0 (0.0)	2 (100.0)
	Methods of engagement adjusted based on feedback N = 2 (5.0)		Refers to RES team making adjustments on a regular basis based on interactions with PAGs to improve support for PPs	0	2	0	2
			Total	0 (0.0)	2 (100.0)	0 (0.0)	2 (100.0)
	Opinions being welcomed, acknowledged, heard, and respected N = 11 (27.5)		Refers to the positive ways COM and PAG’s feel when they speak up in meetings with RES to voice their opinions or concerns	3	2	6	11
			Total	3 (27.3)	2 (18.2)	6 (54.5)	11 (100.0)
	PIs being responsive to requests from PP N = 1 (2.5)		Refers to being responsive	0	1	0	1
			Total	0 (0.0)	1 (100.0)	0 (0.0)	1 (100.0)
	PP active in KT N = 1 (2.5)		Refers to how PP’s participate in KT (presentations)	0	1	0	1
			Total	0 (0.0)	1 (100.0)	0 (0.0)	1 (100.0)
	PP feeling supported N = 2 (5.0)		Refers to feeling supported to share their perspectives and their thoughts regarding project progress	0	0	2	2
			Total	0 (0.0)	0 (0.0)	2 (100.0)	2 (100.0)
	PP involved early in the project N = 1 (2.5)		Refers to the timeline at which PP’s got involved and knowing their value	0	1	0	1
			Total	0 (0.0)	1 (100.0)	0 (0.0)	1 (100.0)
	Qualities of PP N = 4 (10.0)		Refers to the team, how they are engaged, thoughtful, invested, and loyal to the projects	2	2	0	4
			Total	2 (50.0)	2 (50.0)	0 (0.0)	4 (100.0)
	Researchers and PP strong partnership and understanding, mutual goals N = 1 (2.5)		Refers to the mutual partnership, valuing how one is learning from the other to make the project as strong as possible	0	1	0	1
			Total	0 (0.0)	1 (100.0)	0 (0.0)	1 (100.0)
			Grand total	8 (20.0)	17 (42.5)	15 (37.5)	40 (100.0)

*PAG* Patient-advisory group, *RES* researchers, *COMM* committee members, *PP* patient-partners

**Table 9:** PPEET results: Strategies to improve patient engagement (PE)

Theme n = number of utterances	Subtheme n = number of utterances % of Theme	Sub-subtheme n = number of utterances % of Sub-subtheme	Description	PAG utterances n (%)	RES utterances n (%)	COMM utterances n (%)	Total utterances n (%)
Strategies to improve PE N = 45	Actions taken to ensure supports and mitigate barriers N = 1 (2.2)		Refers to the communication between PAGs and RES to demonstrate where change has been implemented	0	1	0	1
			Total	0 (0.0)	1 (100.0)	0 (0.0)	1 (100.0)
	Allow more flexibility in PP engagements N = 1 (2.2)		Refers to ensuring that PAGs continue to want to actively engage without feeling pressure to engage	0	1	0	1
			Total	0 (0.0)	1 (100.0)	0 (0.0)	1 (100.0)
	Appreciation initiatives N = 1 (2.2)		Refers to measures taken to boost team morale and sense of belonging	0	1	0	1
			Total	0 (0.0)	1 (100.0)	0 (0.0)	1 (100.0)
	Appropriate renumeration and other incentives N = 4 (8.9)		Refers to ensuring that PAGs are adequately remunerated given the time and effort they are putting in, and considering the familial situation they’re dealing with	0	2	2	4
			Total	0 (0.0)	2 (50.0)	2 (50.0)	4 (100.0)
	Central database of to dos for PP to make sure no one is overwhelmed with assignments N = 1 (2.2)		Refers to ways to mitigate the feeling of being burdened with work from CB	0	1	0	1
			Total	0 (0.0)	1 (100.0)	0 (0.0)	1 (100.0)
	Clarity on goals, outputs and contributions N = 10 (22.2)		Refers to more clearly informing PAGs and COMM what the goals are, and being more transparent with expectations and timelines	1	5	4	10
			Total	1 (10.0)	5 (50.0)	4 (40.0)	10 (100.0)
	Clearer goals re meetings N = 2 (4.4)		Refers to being clearer earlier on before the meetings so COMMs can get an idea of how their input can be useful	0	0	2	2
			Total	0 (0.0)	0 (0.0)	2 (100.0)	2 (100.0)
	Crosslinkages between committees N = 1 (2.2)		Refers to ideas regarding how engagement can be increased without adding extra hours to the schedule by combining committee goals/research projects	0	1	0	1
			Total	0 (0.0)	1 (100.0)	0 (0.0)	1 (100.0)
	Ensure balance between PP voices N = 2 (4.4)		Refers to making sure that even those who do not speak as often can still make meaningful contributions to the projects	0	2	0	2
			Total	0 (0.0)	2 (100.0)	0 (0.0)	2 (100.0)
	Finding balance between parent partners and youth partners’ voices N = 1 (2.2)		Refers to expanding and diversifying the participants so that one type of voice doesn’t overpower another	0	0	1	1
			Total	0 (0.0)	0 (0.0)	1 (100.0)	1 (100.0)
	Inperson meetings N = 1 (2.2)		Refers to the desire to meet in person	0	1	0	1
			Total	0 (0.0)	1 (100.0)	0 (0.0)	1 (100.0)
	Keep up with promises to give feedback N = 2 (4.4)		Refers to providing feedback on the impact of PAGs throughout the project (when ideas implemented) and also at the end once project results are analysed	0	0	2	2
			Total	0 (0.0)	0 (0.0)	2 (100.0)	2 (100.0)
	Making sure to act on all recommendations N = 2 (4.4)		Refers to acting in one way or another on recommendations given by PAGs	0	0	2	2
			Total	0 (0.0)	0 (0.0)	2 (100.0)	2 (100.0)
	Managing expectations N = 1 (2.2)		Refers to ensuring PAGs and COMs are aware of limitations of the “research world”	0	0	1	1
			Total	0 (0.0)	0 (0.0)	1 (100.0)	1 (100.0)
	PP on REB reviews N = 1 (2.2)		Refers to needing PPs at the stage of REB and not only after the project has been approved	1	0	0	1
			Total	1 (100.0)	0 (0.0)	0 (0.0)	1 (100.0)
	Recruit more diverse members N = 7 (15.6)		Refers to the desire to recruit more diverse members as a way to reflect the health care system and get input from underrepresented groups	1	3	3	7
			Total	1 (14.3)	3 (42.9)	3 (42.9)	7 (100.0)
	Redirect funding to better support PPs in different initiatives N = 1 (2.2)		Refers to the possibility of using funding towards outreach projects as a way to obtain more inclusive perspectives	0	1	0	1
			Total	0 (0.0)	1 (100.0)	0 (0.0)	1 (100.0)
	Strengthen communication channels N = 5 (11.1)		Refers to obtaining feedback in many ways rather than just meetings, for example through emails or one on one meetings	0	3	2	5
			Total	0 (0.0)	3 (60.0)	2 (40.0)	5 (100.0)
	Team building exercise N = 1 (2.2)		Refers to methods that can be implemented to improve team morale, coffee meetings, thank-you cards, to show greater appreciation for their work	0	1	0	1
			Total	0 (0.0)	1 (100.0)	0 (0.0)	1 (100.0)
			Grand total	3 (6.7)	23 (51.1)	19 (42.2)	45 (100.0)

*PAG* Patient-advisory group, *RES* researchers, *COMM* committee members, *PP* patient-partners

Advantages of patient engagement included research project benefits (valuable input making research more relevant and representative of end users’ needs, improvement of recruitment, methodology, relevance of knowledge translation and dissemination, keeping research team on track, and pilot testing and modification to the protocol); learning and having a voice (having a chance to advocate for self and peers, learning about others’ perspectives and about strategies for patient-oriented research, and adapting to changes in interactions related to the pandemic); and connections (collaboration opportunities, close contact with research team, and bridging gaps between people involved). These benefits were most reported by researchers and committee members (92.5% of statements), and less by patient-partners (7.3%). Most patient-partners’ reported benefits were related to increasing the research relevance and representativeness (50% of patient-partners’ utterances), while researchers mainly reported on methodological/recruitment and knowledge mobilization benefits (62.5% of researchers’ utterances).

Barriers to patient engagement were diverse. They were mainly identified by researchers (59.3% of statements) and committee members (29.6%), and less by patient-partners on projects (11.1%). Most reported limitations were group homogeneity, lack of clarity regarding roles, deciding what perspectives from patient-partners are to be incorporated and how, as well as scheduling and organizing meetings that suit everyone’s availabilities.

In terms of facilitators, patient-partners were more outspoken (20.0% of statements) than in other sections of the PPEET (6.7–11.1%). The main facilitator identified by all groups was related to effective communication strategies. More specifically, optimal meeting organization and planning was reported as an important enabler. Furthermore, it emerged that one main facilitator is ensuring that opinions are being welcomed, acknowledged, heard, and respected.

Lastly, nineteen (n = 19) different strategies to improve patient engagement were conveyed by all key stakeholder groups. One commonly reported solution by committee members and researchers was to promote the clarity on goals, outputs, and contributions by being transparent with expectations and timelines (22.2% of utterances). Moreover, recruiting more diverse members to enhance the existing patient-partner group’s heterogeneity was proposed by all groups, including patient-partners (15.6%). Further strengthening communication channels and ensuring appropriate renumeration/other incentives were reported as other possible strategies by researchers and committee members.

## Discussion

Our objectives were to measure patient engagement longitudinally as the research projects evolved through their cycle in the CHILD-BRIGHT Network, and to explore the perceived benefits, barriers and facilitators and overall satisfaction with patient engagement from the perspectives of the different stakeholder groups in the Network. Our process began with measuring patient engagement using the CBPR questionnaire, where it was applied yearly over a 3-years period and was suitable to define the research processes and activities of the research project that Network members were involved in, to appraise the perceived level of the partnership, and the type of trust in this partnership. Understanding how partnerships are formed and evolve provided us with insights into the dynamics of collaboration within the Network. Assessing partnership development helped us uncovering potential factors that contribute to successful and sustainable research partnerships. In Year 3, we aimed to deepen our understanding of patient engagement and implemented the PPEET in addition to the CBPR questionnaire.

Overall, as indicated by all the key stakeholder groups, including researchers, committee members, patient-partners, and youth advisors, data suggest that over the 3-years period we have been effectively implementing patient-oriented research strategies. Our findings show that patient-partners were satisfied with their level of engagement in the Network’s research and governance, and the quality of patient engagement was highly rated across the different stakeholder groups. Notably, in Year 1, our findings show a large proportion of stakeholders expressing satisfaction with their level of involvement in the project, which remained robust in subsequent years. Importantly, stakeholders highlighted feeling comfortable sharing their opinions when partners came together, showcasing positive collaborative dynamics. More specifically, in appraising the partnership, we noted that with time, patient-partners gained comfort in sharing their opinions. This was shown by a more prevalent agreement among respondents in Years 2 (91%) and 3 (92%) compared to Year 1 (74%) on the CBPR questionnaire. This encouraging finding is further supported by what we determined using the PPEET, where more than 80% of patient-partners (81.3–100%) reported being able to express their views, felt that they were being heard, that a wide range of topics were discussed in meetings, and that they contributed to a broad range of perspectives in these discussions. To the contrary, previous research in the fields of cancer and low-back pain indicated that patient-partners felt that they were not being listened to [22–24] and reported feeling excluded from regular interactions with the research team [22, 25]. We propose that by ensuring regular communications with our patient-partners and researchers on findings from our Network-wide measurement efforts, offering training opportunities and feedback to the teams, and enabling patient-partners to share experiences with our parent peer mentor, the patient engagement was supported and strengthened.

Moreover, throughout the study period, a substantial majority of patient-partners viewed the project as a "true partnership”, suggesting a widespread belief in the collaborative and equitable nature of relationships among stakeholders. Furthermore, endorsement of critical reflective trust, characterized by an environment where mistakes can be discussed and resolved, increased over the years. Our findings indicated that the highest endorsement was observed in Year 3, revealing a growing level of trust among stakeholders. Similarly, our study determined that researchers consistently appraised the partnership positively across various items and the highest level of trust endorsement was most prevalent in Year 3, indicating a strengthening of trust over time. Overall results from the CBPR questionnaire suggest a positive trajectory in stakeholder involvement, partnership dynamics, and trust within the CHILD-BRIGHT Network. The consistent positive appraisals and the increasing endorsement of critical reflective trust underscore the success of the collaborative efforts and the establishment of a true partnership within the Network.

Additionally, our findings highlighted a fundamental facilitator of patient-oriented research, specifically in the realm of communication strategies. This is in line with previous findings from another research network in the field of rheumatologic conditions [26], where communication was identified as the key facilitator. Similarly, within one of the 13 research projects in our Network (BRIGHT Coaching, Theme 3: Service delivery redesign to address gaps in service), researchers and patient-partners recognized facilitating communication as a crucial factor in enabling patient engagement [8]. Specifically, the BRIGHT Coaching group administered the PPEET midway within the randomized trial to members of their research team (researchers and patient-partners). In this “project-specific” patient engagement evaluation, fewer patient-partners agreed with statements related to communication, sharing views and perspectives (on the PPEET) than in the overall Network’s evaluation in 2021 (50–74% vs. 83.3–100% “Strongly agree”). Following the 2019 project-specific evaluation, we have implemented strategies to improve patient engagement, particularly in this period of data collection. These included increasing accountability of how and what patient-partners’ feedback was being incorporated; involving patient-partners more actively in discussing what factors should be considered as important in data analysis and developing plans for knowledge dissemination of results; implementing a shared directory of ongoing and planned tasks involving patient-partners that allows team members to view task assignments and avoid surcharging patient-partners; and offering more time/date options for virtual meetings. We suggest that these measures had a positive impact on patient engagement by fostering a more inclusive and transparent collaboration and addressing communication-related concerns.

In our longitudinal evaluation of patient engagement, we identified that the lack of role clarity was one of the main reported barriers. Another Canadian network in the field of cardiovascular research also found that the lack of role clarity was one of the emerging barriers to patient engagement [27]. In our investigation, the most commonly proposed solution to improve patient engagement was to clearly inform all team members of the goals of their group (e.g., committee, project), their roles, expectations, and timelines. We acknowledge that these factors are not static and can change over time. For instance, in using the CBPR questionnaire, we confirmed that patient-partners’ engagement in the research process evolved with time, where they were mostly involved in processes related to project development, recruitment and data collection in the first 2 years and progressed to mainly disseminating findings in Year 3. Within the CHILD-BRIGHT Network, several projects have now identified an individual or individuals (e.g., patient-partner, research coordinator, and/or researcher) who connects regularly with their patient-partner advisory group to clarify their roles as the research project evolves.

Our longitudinal evaluations have also demonstrated other areas for improvement. Some respondents highlighted the need to enhance the diversity of our patient-partner members. Efforts are underway to recruit more diverse members into the Network to ensure we are getting input from underrepresented groups. One strategy that is being implemented is the creation of the Equity, Diversity, Inclusion, Decolonization & Indigenization (EDI-DI) Program. The EDI-DI program aims to ensure all voices, bodies, and experiences are included in all aspects of CHILD-BRIGHT's work by deploying initiatives (e.g., consultation, training) based on EDI-DI principles and embedding them in the Network’s programs and governance structure. We anticipate that the EDI-DI program can transform the landscape of patient engagement by fostering inclusivity, cultural competence, and equity, enhance the experience of patients involved in the Network and positively contribute to the effectiveness and relevance of patient engagement initiatives. Moreover, future evaluations of patient engagement within this context can benefit from considering the unique impacts and outcomes across diverse communities.

One positive finding from our longitudinal evaluations is that over 80% of patient-partners reported that their group (research project, program committee) was on the right path to achieving its objectives and reported having high confidence that their input was used and made a difference in the work related to their group. In fact, ‘research project-related benefits’ was the most frequently mentioned in the patient engagement benefits sub-theme. Specifically, Network members indicated that patient engagement was important in improving the impacts of knowledge translation and dissemination; as well as in providing valuable input to ensure that the research was more relevant and representative of end-users’ needs. This is consistent with previous research on the benefits of patient engagement [4, 5, 28–30] and the CIHR SPOR mandate [1].

A working group of patient-partners, researchers, trainees, and staff oversaw the implementation and analysis of the patient engagement measurement strategies. Results were carefully reviewed, and our Citizen Engagement Council and our National Youth advisory panel developed tipsheets for researchers [9], parent-partners [10] and youth-partners [11] that describe the ‘how to’ for successful patient-oriented research. We also summarized the key findings of this study on a blog highlighting key lessons learned in a visual summary format and full report [12]. As mentioned above, the Network adjusted its efforts in support of patient engagement over time. We also conducted interviews with researchers and patient-partners to further understand barriers and facilitators to patient-oriented research, to inform our engagement strategies moving forward [7].

This study has limitations, particularly with a relatively modest response rate (average across all evaluations: 31.9%), as not all Network members participated in the patient engagement survey(s) at every measurement timepoint. In addition, we have observed a decrease in the response rate on the CBPR (61.4%, 30.2%, 14.2% for Years, 1, 2, and 3), which could be the result of using another tool in Year 3 (PPEET) and the COVID-19 pandemic. While efforts were made to encourage participation, the varying response rates may impact the generalizability of the findings. Moreover, it was not possible for us to conduct a single-subject repeated measures design to confirm changes in individual perspectives related to the patient engagement process, satisfaction with the experience, and its impact. Nevertheless, we determined that on average, there was a high and stable satisfaction level with patient engagement processes. Importantly, our measurements indicated an improvement in communication strategies and patient-partners’ comfort level in sharing their opinions over time.

The most important lessons learned from our patient engagement evaluation journey include: (i) it is feasible to evaluate patient engagement in a large Network both cross-sectionally and over time, (ii) there are several standardized measures available to carry out these evaluations and these could be used jointly to complement findings, and (iii) these periodic assessments are critical in enabling the Network to reflect on the best practices of patient-partnership and ways to optimally support authentic engagement practices [7].

## Conclusion

In conducting our evaluation approaches over time, we determined that patient engagement was positively rated. Several areas were highlighted for some improvement, which allowed the Network to develop strategies to support authentic engagement more optimally. These results are encouraging given that we partner with members across a large, nation-wide Network, where connections are primarily virtual, and most members were novices to the patient-oriented research process at the onset of the Network’s activities.

### Supplementary Information

**Additional file 1.** S1: Guidance for reporting involvement of patients and the public (GRIPP2) short form. S2: PPEET response frequencies per stakeholder group. S3: PPEET qualitative response: Most salient utterances defining emerging themes and subthemes.

## Data Availability

The datasets used and/or analyzed during the current study are available from the corresponding author on reasonable request.
