# Supplementary material for: Lessons learned in measuring patient engagement in a Canada-wide childhood disability network
Source: Res Involv Engagem. 2024 Feb 7;10:18. doi: 10.1186/s40900-024-00551-9 (PMC10851468; doi:10.1186/s40900-024-00551-9)
Supplement: Supplementary file 1 — Additional file 1. S1: Guidance for reporting involvement of patients and the public (GRIPP2) short form. S2: PPEET response frequencies per stakeholder group. S3: PPEET qualitative response: Most salient utterances defining emerging themes and subthemes. [file 40900_2024_551_MOESM1_ESM.docx]

**Supplementary Material 1** Guidance for reporting involvement of patients and the public (GRIPP2) short form

| **Section and topic** | **Item** | **Reported on page No** |
| --- | --- | --- |
| 1: Aim | Report the aim of the PPI in the study. | P. 6 |
| 2: Methods | Provide a clear description of the methods used for PPI in the study. | P. 7-13 |
| 3: Study results | Outcomes – report the results of the PPI in the study, including both positive and negative outcomes | P. 13-19 |
| 4. Discussion and conclusion | Outcomes – comment on the extent to which PPI influenced the study overall. Describe positive and negative effects. | P.19-23 |
| 5. Reflections/critical perspective | Comment critically on the study, reflecting on the things that went well and those that did not, so others can learn from this experience. | P. 23 |
| PPI = Patient and Public Engagement | | |

**Supplementary Material 2** PPEET response frequencies per stakeholder group

1. Committee members

|  | **Strongly disagree**  **n (%)** | **Disagree**  **n (%)** | **Neither agree nor disagree**  **n (%)** | **Agree**  **n (%)** | **Strongly Agree**  **n (%)** |
| --- | --- | --- | --- | --- | --- |
| **COMMUNICATION & SUPPORTS FOR PARTICIPATION** | | | | | |
| *I have a clear understanding of the purpose of the participation of patient partners in the _____ committee.* | - | 1 (4.2) | 1 (4.2) | 8 (33.3) | 14 (58.3) |
| *Parent-partners have the supports they need to participate (e.g. meeting time and methods, travel, accommodations, etc.).* | - | - | 3 (12.5) | 8 (33.3) | 13 (54.2) |
| *Parent-partners have enough information to contribute to the topic(s) being discussed.* | - | 2 (8.3) | 1 (4.2) | 10 (41.7) | 11 (45.8) |
| **SHARING VIEWS & PERSPECTIVES** | | | | | |
| *Parent-partners are able to express their views freely.* | - | - | 1 (4.2) | 3 (12.5) | 20 (83.3) |
| *Parent-partners feel that their views are being heard.* | - | - | 4 (16.7) | 9 (37.5) | 11 (45.8) |
| *The individuals participating in the _____ committee bring forward a broad range of perspectives on the discussion topics.* | - | 1 (4.2) | 3 (12.5) | 10 (41.7) | 10 (41.7) |
| **IMPACTS & INFLUENCES of ENGAGEMENT INITIATIVE** | | | | | |
| *I think that the _____ committee has achieved or is on the right path to achieve its objectives.* | - | 3 (12.5) | 3 (12.5) | 14 (58.3) | 4 (16.7) |
| *I am confident the input provided by patient-partners is used by the ___ committee of the CHILD-BRIGHT Network.* | - | - | 1 (4.2) | 7 (29.2) | 16 (66.7) |
| *I think that the input provided through patient-partners will make a difference in the work of the _____ committee.* | - | - | 1 (4.2) | 2 (8.3) | 21 (87.5) |
| **FINAL THOUGHTS** | | | | | |
| *As a result of my involvement in the _____ committee, I am better informed about SPOR.* | - | 3 (12.5) | 1 (4.2) | 9 (37.5) | 11 (45.8) |
| *Overall, I am satisfied with the patient-partner contributions on the ___ committee.* | - | - | 2 (8.7) | 9 (39.1) | 12 (52.2) |
| *The input from patient-partners on the _____ committee is a valuable resource.* | - | - | 2 (8.3) | 2 (8.3) | 20 (83.3) |

Note. Color representation: White (<25% response frequency); Light (25-49%); Medium (50-74%); Dark (<75%).

1. Patient-partners on committees

|  | **Strongly disagree**  **n (%)** | **Disagree**  **n (%)** | **Neither agree nor disagree**  **n (%)** | **Agree**  **n (%)** | **Strongly Agree**  **n (%)** |
| --- | --- | --- | --- | --- | --- |
| **COMMUNICATION & SUPPORTS FOR PARTICIPATION** | | | | | |
| *I have a clear understanding of the purpose of my contributions as a patient-partner on the _____ committee.* | - | - | 1 (16.7) | 2 (33.3) | 3 (50.0) |
| *The supports I need to participate are available (e.g. times and methods [GoToMeetings, phone] of meetings, enough time of notice prior to meetings, travel, accommodations, meals).* | - | - | - | 2 (33.3) | 4 (66.7) |
| *I have enough information to contribute to the topic(s) being discussed.* | - | - | 1 (16.7) | 3 (50.0) | 2 (33.3) |
| *I am satisfied with the compensation I received for my role on the _____ committee.* | - | - | 2 (33.3) | 1 (16.7) | 3 (50.0) |
| *The committee is generally prepared for meetings (e.g. agenda/questions for the upcoming meeting and minutes of previous meeting are provided ahead to time to review, timelines are outlined, etc.).* | - | - | - | 2 (33.3) | 4 (66.7) |
| *I receive information with enough time to read and respond within the context of my work/life schedule.* | - | - | - | 5 (83.3) | 1 (16.7) |
| **SHARING VIEWS & PERSPECTIVES** | | | | | |
| *I am able to express their views freely.* | - | - | - | 3 (50.0) | 3 (50.0) |
| *I feel that their views are heard.* | - | - | - | 3 (50.0) | 3 (50.0) |
| *A wide range of views are shared on the topics discussed during our meetings and activities.* | - | - | 1 (16.7) | 2 (33.3) | 3 (50.0) |
| *The individuals participating as patient-partners in the _____ committee bring forward a broad range of perspectives of the discussion topics.* | - | - | 1 (16.7) | 2 (33.3) | 3 (50.0) |
| **IMPACTS & INFLUENCES of ENGAGEMENT INITIATIVE** | | | | | |
| *I think that the _____ committee has achieved or is on the right path to achieve its objectives (mandate of the committee).* | - | - | - | 5 (83.3) | 1 (16.7) |
| *I am confident my input provided as a patient-partner is used by the _____ committee.* | - | - | 1 (16.7) | 3 (50.0) | 2 (33.3) |
| *I think that the input provided by patient-partners in general will make a difference in the work of the _____ committee.* | - | - | 1 (20.0) | 2 (40.0) | 2 (40.0) |
| **FINAL THOUGHTS** | | | | | |
| *As a result of my participation as a patient-partner on the _____ committee, I am better information about SPOR (i.e. methods employed by the researchers to involve and work in collaboration with patient-partners on the committee).* | - | - | - | 2 (33.3) | 3 (50.0) |
| *Overall, I am satisfied with my role as patient-partner on the ___ committee.* | - | - | 1 (20.0) | 2 (40.0) | 2 (40.0) |
| *My role as a patient-partner on the ___ committee is a good use of my time.* | - | - | 1 (20.0) | 2 (40.0) | 2 (40.0) |

Note. Color representation: White (<25% response frequency); Light (25-49%); Medium (50-74%); Dark (<75%).

1. Patient-partners on projects

|  | **Strongly disagree**  **n (%)** | **Disagree**  **n (%)** | **Neither agree nor disagree**  **n (%)** | **Agree**  **n (%)** | **Strongly Agree**  **n (%)** |
| --- | --- | --- | --- | --- | --- |
| **COMMUNICATION & SUPPORTS FOR PARTICIPATION** | | | | | |
| *I have a clear understanding of the purpose of my contributions as a patient-partner on the _____ project.* | - | - | 2 (11.8) | 7 (41.2) | 8 (47.1) |
| *The supports I need to participate are available (e.g. times and methods [GoToMeetings, phone] of meetings, enough time of notice prior to meetings, travel, accommodations, meals).* | - | - | 1 (5.9) | 10 (58.8) | 6 (35.3) |
| *I have enough information to contribute to the topic(s) being discussed.* | - | - | 1 (5.9) | 10 (58.8) | 6 (35.3) |
| *I am satisfied with the compensation I received for my role on the _____ project.* | - | - | 1 (5.9) | 9 (52.9) | 7 (41.2) |
| *The research team is generally prepared for meetings (e.g. agenda/questions for the upcoming meeting and minutes of previous meeting are provided ahead to time to review, timelines are outlined, etc.).* | - | - | 1 (5.9) | 5 (29.4) | 11 (64.7) |
| *I receive information with enough time to read and respond within the context of my work/life schedule.* | - | - | - | 11 (64.7) | 6 (35.3) |
| **SHARING VIEWS & PERSPECTIVES** | | | | | |
| *I am able to express their views freely.* | 1 (5.9) | - | - | 4 (23.5) | 12 (70.6) |
| *I feel that their views are heard.* | - | - | - | 7 (41.2) | 10 (58.8) |
| *A wide range of views are shared on the topics discussed during our meetings and activities.* | - | - | 2 (11.8) | 8 (47.1) | 7 (41.2) |
| *The individuals participating as patient-partners in the ____ project bring forward a broad range of perspectives of the discussion topics.* | - | 1 (6.3) | 2 (12.5) | 7 (43.8) | 6 (37.5) |
| **IMPACTS & INFLUENCES of ENGAGEMENT INITIATIVE** | | | | | |
| *I think that the _____ project has achieved or is on the right path to achieve its objectives.* | - | - | 1 (5.9) | 6 (35.3) | 10 (58.8) |
| *I am confident my input provided as a patient-partner is used by the _____ project.* | - | - | 2 (11.8) | 7 (41.2) | 8 (47.1) |
| *I think that the input provided by patient-partners in general will make a difference in the work of the _____ project.* | - | - | 1 (5.9) | 7 (41.2) | 9 (52.9) |
| **FINAL THOUGHTS** | | | | | |
| *As a result of my participation as a patient-partner on the _____ project, I am better information about SPOR (i.e. methods employed by the researchers to involve and work in collaboration with patient-partners on the research project).* | - | - | 2 (12.5) | 6 (37.5) | 8 (50.0) |
| *Overall, I am satisfied with my role as patient-partner on the _____ project.* | - | - | 2 (12.5) | 6 (37.5) | 8 (50.0) |
| *My role as a patient-partner on the _____ project is a good use of my time.* | - | - | 1 (6.3) | 8 (50.0) | 7 (43.8) |

Note. Color representation: White (<25% response frequency); Light (25-49%); Medium (50-74%); Dark (<75%)

1. Researchers

|  | **Strongly disagree**  **n (%)** | **Disagree**  **n (%)** | **Neither agree nor disagree**  **n (%)** | **Agree**  **n (%)** | **Strongly Agree**  **n (%)** |
| --- | --- | --- | --- | --- | --- |
| **COMMUNICATION & SUPPORTS FOR PARTICIPATION** | | | | | |
| *I have a clear understanding of the purpose of the parent advisory committee of the _____ project.* | - | - | - | 15 (37.5) | 25 (62.5) |
| *Parent-advisors have the supports they need to participate (e.g. meeting time and methods, travel, accommodations, etc.).* | - | 1 (2.4) | 2 (4.9) | 23 (56.1) | 15 (36.6) |
| *Parent-advisors have enough information to contribute to the topic(s) being discussed.* | - | - | 3 (7.7) | 22 (56.4) | 14 (35.9) |
| **SHARING VIEWS & PERSPECTIVES** | | | | | |
| *Parent-advisors are able to express their views freely.* | - | - | - | 20 (48.8) | 21 (51.2) |
| *Parent-advisors feel that their views are being heard.* | - | - | 4 (10.0) | 20 (50.0) | 16 (40.0) |
| *The individuals participating in the parent advisory committee of the _____ project bring forward a broad range of perspectives on the discussion topics.* | - | 2 (5.0) | 4 (10.0) | 16 (40.0) | 18 (45.0) |
| **IMPACTS & INFLUENCES of ENGAGEMENT INITIATIVE** | | | | | |
| *I think that the _____ project has achieved or is on the right path to achieve its objectives.* | - | 1 (2.4) | 3 (7.3) | 22 (53.7) | 15 (36.6) |
| *I am confident the input provided through the parent-advisory committee is used by the _____ project of the CHILD-BRIGHT Network.* | - | 1 (2.4) | 1 (2.4) | 13 (31.7) | 26 (63.4) |
| *I think that the input provided through the parent-advisory committee in general will make a difference in the work of the _____ project.* | - | - | 2 (4.9) | 9 (22.0) | 30 (73.2) |
| **FINAL THOUGHTS** | | | | | |
| *As a result of my involvement in the _____ project, I am better informed about SPOR.* | - | 1 (2.6) | 4 (10.3) | 13 (33.3) | 21 (53.8) |
| *Overall, I am satisfied with the parent-advisory committee of the _____ project.* | - | - | 4 (10.3) | 14 (35.9) | 21 (53.8) |
| *The parent-advisory committee of the _____ project is a valuable resource.* |  |  | 1 (2.6) | 5 (12.8) | 33 (74.6) |

Note. Color representation: White (<25% response frequency); Light (25-49%); Medium (50-74%); Dark (<75%).

1. Youth on committees

|  | **Strongly disagree**  **n (%)** | **Disagree**  **n (%)** | **Neither agree nor disagree**  **n (%)** | **Agree**  **n (%)** | **Strongly Agree**  **n (%)** |
| --- | --- | --- | --- | --- | --- |
| **COMMUNICATION & SUPPORTS FOR PARTICIPATION** | | | | | |
| *I have a clear understanding of the purpose of my contributions as a youth advisor on the NYAP.* | - | - | - | 1 (25.0) | 3 (75.0) |
| *The supports I need to participate are available (e.g. times and methods [GoToMeetings, phone] of meetings, enough time of notice prior to meetings, travel, accommodations, meals).* | - | - | - | - | 4 (100) |
| *I have enough information to contribute to the topic(s) being discussed.* | - | - | - | - | 4 (100) |
| *I am satisfied with the compensation I received for my role on the NYAP.* | - | - | - | - | 4 (100) |
| *The panel is generally prepared for meetings (e.g. agenda/questions for the upcoming meeting and minutes of previous meeting are provided ahead to time to review, timelines are outlined, etc.).* | - | - | - | - | 4 (100) |
| *I receive information with enough time to read and respond within the context of my work/life schedule.* | - | - | - | - | 4 (100) |
| **SHARING VIEWS & PERSPECTIVES** | | | | | |
| *I am able to express their views freely.* | - | - | - | - | 4 (100) |
| *I feel that their views, opinions and contributions are heard and respected.* | - | - | - | - | 4 (100) |
| *A wide range of views are shared on the topics discussed during our meetings and activities.* | - | - | - | - | 4 (100) |
| *The individuals participating as youth advisors on the NYAP bring forward a broad range of perspectives of the discussion topics.* | - | - | - | - | 4 (100) |
| **IMPACTS & INFLUENCES of ENGAGEMENT INITIATIVE** | | | | | |
| *I think that the NYAP has achieved or is on the right path to achieve its objectives (mandate of the panel).* | - | - | - | 3 (75.0) | 1 (25.0) |
| *I am confident my input as youth advisor is used by the NYAP.* | - | - | - | - | 4 (100) |
| *I think that the input provided by youth advisors in general will make a difference in the work of the NYAP.* | - | - | - | - | 4 (100) |
| **FINAL THOUGHTS** | | | | | |
| *As a result of my participation as a youth advisor on the NYAP, I am better information about SPOR (i.e. methods employed by the researchers to involve and work in collaboration with patient-partners on the panel and in the network).* | - | - | - | - | 4 (100) |
| *Overall, I am satisfied with my role as youth advisor on the NYAP.* | - | - | - | - | 4 (100) |
| *My role as a youth advisor on the NYAP is a good use of my time.* | - | - | - | - | 4 (100) |

Note. Color representation: White (<25% response frequency); Light (25-49%); Medium (50-74%); Dark (<75%).

**Supplementary Material 3** PPEET qualitative response: Most salient utterances defining emerging themes and subthemes.

**Benefits/advantages of patient engagement: Most salient utterances**

| **Theme** | **Sub-theme** | **Examples of participants’ quotes** |
| --- | --- | --- |
| **Connections** | **Bridging gaps between people involved** | **COMM:**  “Bridging the gaps between all people involved” |
|  | **Close communication with research team** | **PAG: “**Being on the ground floor, and having a direct line to the research team. Being able to ask questions and give input. Better grasp of how research works. Able to help with recruitment” |
|  | **Collaboration opportunities and human connections** | **PAG:**    **“**It's been a good experience to collaborate with many great people from different backgrounds.”    “I enjoyed the being in contact with a fellow parent regarding ideas for better engagement of partners.” |
| **Learning and voicing** | **For PP- Having a chance to advocate for self and peers** | **COMM:**    **“**I truly appreciate the chance to advocate for myself and future participants  Learning about patient-oriented research, and providing a voice for youth with disabilities”    “Learning about patient-oriented research strategies, and providing a voice for those affected by brain-based disabilities.”    “Learning about patient-oriented research strategies, and providing a voice for those affected by brain-based disabilities.” |
|  | **Learning about others’ perspectives and CEC mandate** | **COMM:**  “Learning other people's perspectives. Learning about the CEC's mandate.”    **RES:**  **“**The biggest advantage is that the PFAC is that the work, and I hope the end product, is meaningful to patients and families. Moreover, it is so much more fun to work in partnership. Great opportunities for learning for everyone.” |
|  | **Learning about SPOR** | **COMM:**  **“**Learning about patient-oriented research strategies and providing a voice for those affected by brain-based disabilities.”    **RES:**  **“**they ensure that our research approach and our intervention are parent-centred and user-friendly. All our materials are vetted to ensure that they are understandable and helpful. It has been such a wonderful experience working in partnership with our parent advisors, to learn more about the processes and benefits of patient-oriented research.”    **“**The biggest advantage is that the PFAC is that the work, and I hope the end product, is meaningful to patients and families. Moreover, it is so much more fun to work in partnership. Great opportunities for learning for everyone.” |
|  | **Quick adaptation to the changes in interactions related to the pandemic** | **RES:**    **“**Obviously COVID 19 has had an impact on research and has drastically changed how patient-partner interactions happen, but I think under the circumstances everyone is doing their best to continue on with this research in a meaningful way.” |
| **Research project benefits** | **Improved recruitment and or research methodology** | **RES:**  **“**They also helped with pilot testing the questionnaires to fix errors and wording, explain the reasons for why questions are being asked, and overall improved the experience of participants in the study. They are invested in ensuring the success of the project, in which they will play an important role in sharing their social media connections to other organizations and family partner groups to advertise the recruitment of the study.”    **“**Having parent perspectives is invaluable to the project with regard to program development and various aspects of study design and implementation. We all have our own expertise and lenses through which we view this project and parents' views are essential to understanding (and improving) how the program and study will be experienced by other parents.”    “The PFAC has had a significant influence in enhancing the work of the READYorNot project. For example, they have been involved with the RCT procedures, training and other supporting materials which includes the testimonial-style recruitment videos to highlight the importance of participating in the project and reference handouts for study participants.” |
|  | **Improved relevance of knowledge translation and dissemination** | **PAG:**    **“**The content has direct feedback from parents, helps shape the language and the approach in a way that parents can relate to.”    **COMM:**    “The patient-partner comes with a lot of experience and they enhance work of the committee. I think one advantage for us is that, due to their sustained engagement in the committee, they've been involved in additional network committees and initiatives (e.g. conference programming committee, symposia, etc). Also, the patient-partner provides us with valuable anecdotes of how parent/participants want to receive information. Overall, their presence is very grounding.”    **RES:**    **“**Having a different perspective on the project, the development and the RCT strategies, materials, tools. They are giving us "their" vision which is very important. Knowing what the "user" or the families of the users expect from the App and the research makes it less likely to fail” |
|  | **Keeping the research team on track** | **COMM:**    **“**I have to be honest, I'm not entirely sure I understand the mandate of the research committee...but it might be because I wasn't involved from the very beginning and several research projects were already in flight by the time I joined, therefore the committee meetings have primarily been about "touching base". I have been trying to bring forward some initiatives to the research committee around "data reuse", but would say that it has not been entirely successful - it's perhaps not a topic of great interest to the committee and/or my strategy has been a bit unfocused (and I've not been able to move quickly on any of the ideas presented). However, in recent months, we've established a revised task force of the research committee with the purpose of developing a blog series about research data reuse - we have two patient partners who joined in the last 6 months and things are really starting to gel with this task force. Having the patient partners engaged in the task force has really helped us focus our efforts and infused a level of "purpose" that we really didn't have before - their input is providing a new direction for the initiative and keeps on on track (e.g. we now have a timeline and deliverables).” |
|  | **Pilot testing and modifications to the protocol** | **RES:**    **“**They also helped with pilot testing the questionnaires to fix errors and wording, explain the reasons for why questions are being asked, and overall improved the experience of participants in the study. They are invested in ensuring the success of the project, in which they will play an important role in sharing their social media connections to other organizations and family partner groups to advertise the recruitment of the study.”    **“**They also helped with pilot testing the questionnaires to fix errors and wording, explain the reasons for why questions are being asked, and overall improved the experience of participants in the study. They are invested in ensuring the success of the project, in which they will play an important role in sharing their social media connections to other organizations and family partner groups to advertise the recruitment of the study” |
|  | **PP providing input on hot to better engage patients** | **COMM:**    **“**There has been a valuable partnership with patient-partners in the Training Committee, and they consistently have provided input about how to engage with family partners across all initiatives in the CHILD-BRIGHT Network. They have also provided insights into potential gaps that could be addressed by the network. For example, we are currently planning the Summer Learning Series to learn about strategies in engaging with youth in research.” |
|  | **PP steering actions right from the start** | **COMM:**    **“**They truly steer what we are doing, not just providing approval. This means it is oriented to the patient experiences from the start.” |
|  | **Understanding gaps in knowledge that needs to be filled** | **COMM:**    **“**Diversity of opinions and perspectives. A better understanding of the gaps in knowledge that we have to fill.”    **“**There has been a valuable partnership with patient-partners in the Training Committee, and they consistently have provided input about how to engage with family partners across all initiatives in the CHILD-BRIGHT Network. They have also provided insights into potential gaps that could be addressed by the network. For example, we are currently planning the Summer Learning Series to learn about strategies in engaging with youth in research.” |
|  | **Valuable input making research more relevant, and representative of end users’ needs** | **PAG:**    **“**I have been supported in attending the EPIQ conference two years in a row and this has been valuable. I think the way parent engagement is viewed and valued is on the rise and I saw an increase in positive engagement of parents at the conference from 2019 to 2020.”    **COMM:**    **“**They all have lots of experience and a good bit of wisdom to offer. They're also very committed to the network's success. In particular they bring both an inside understanding of the network and an outside perspective. Most are also in touch with many other patient-partners and bring what they've heard and learned from them.”    **RES:**  **“**The PFAC has been a strong partner in this project, in which their feedback has helped to enhance the design and execution of the project. We have welcomed feedback and input from PFAC throughout various stages of the project. They have been integral in co-creating the App to ensure that youth participants would have a meaningful experience. They have also provided feedback for the language and wording of various materials, including testimonial-style videos for recruitment and reference handouts. A challenge that we experienced is the decision-making process of whether perspectives from PFAC are incorporated into the project. For example, we had to outline boundaries around what the App "can" and "cannot do". In order to overcome this challenge, we discussed about being transparent by sharing with PFAC members about how the feedback was incorporated and our rationale for when their feedback was not incorporated.” |

Legend: Committee member (COMM); Member of the parent-group advisory (PAG); Researcher (RES).

**Barriers to patient engagement: Most salient utterances**

| **Theme** | **Sub-theme** | **Examples of participants’ quotes** |
| --- | --- | --- |
| **Ambiguity in goals** |  | **COMM:**  “Sometimes, the goals that are set out and are quite ambitious. It must be acknowledged that when a goal is achieved such as the implementation of a program an evaluation of the program/goal will be needed.” |
| **Availabilities of PP** |  | **RES:**  **“**The transition from development to data collection, where the type of engagement changed significantly. A recommendation would be to plan for how the roles of our committee changes over the course of project, to better communicate with our parents and transition between phases.”    **“**Parents are not always available, as they have a lot on their plate and may at times be experiencing greater stresses. It is important to have a large enough parent advisory team, so that a subset can disconnect when necessary and not feel the pressure of responding and interacting with the team at all times. It is key to allow the flexibility for parents to participate when possible.” |
| **Compensation issues** |  | **RES: “**There was a long delay with re-imbursement for one family's attendance at t CHILD-BRIGHT annual meeting - not sure whether this was due to an oversight on my part re: paperwork.” |
| **Complexity of protocols and research steps** |  | **COMM:**  “I know a lot less than the others about many aspects of research--the complexity of protocols, all the potential difficulties of getting ethics approval, budgetary arrangements, etc. Sometimes it's hard to know how much I need to know or should know about x.”    **RES:**  **“**feedback from our personal check-in calls with PFAC members: Sometimes we give too many details (e.g., showing an elaborate diagram of recruitment sites). Instead, have a balance with the amount of information we share of what is needed to know for PFAC members.” |
| **Concurrent commitments impending engagement** |  | **RES:**  **“**A challenge is to attract new PFAC members, as three current PFAC members have indicated to step back due to other commitments (which is fully understandable).”    **“**The partners who are able to give time to this project are able to express views. Our concern from day one continues: that the seldom heard are just that. I know this is a concern from Child Bright too, however, we've been raising the issue for many years (long before child bright) and we are struggling to find solutions, although have made some inroads re translation. It's just something to really keep at the top of the agenda.” |
| **Deciding what perspectives from PP are to be incorporated and how** |  | **COMM:**  **“**It's not always practical to implement all the advise from the patient-partner due to institutional regulations, for example. But this is also a positive, as we can take it to the institutions and question certain regulations/advocate for change.”    **RES:**  **“**The PFAC has been a strong partner in this project, in which their feedback has helped to enhance the design and execution of the project. We have welcomed feedback and input from PFAC throughout various stages of the project. They have been integral in co-creating the App to ensure that youth participants would have a meaningful experience. They have also provided feedback for the language and wording of various materials, including testimonial-style videos for recruitment and reference handouts. A challenge that we experienced is the decision-making process of whether perspectives from PFAC are incorporated into the project. For example, we had to outline boundaries around what the App "can" and "cannot do". In order to overcome this challenge, we discussed about being transparent by sharing with PFAC members about how the feedback was incorporated and our rationale for when their feedback was not incorporated.”    “Acting on patient-partner feedback within the integrated study team is fairly seamless currently, but there is a benefit of documenting those actions. Though they might seem small (e.g. having a point person to touch base with first thing in the morning on camp days) they can have a significant impact on the patient experience. Documentation will allow us to transfer those learnings if staff change and to carry those things forward in future projects.” |
| **Difficulty understanding the science involved and the research process** |  | **PAG:**  **“**Sometimes it is challenging to understand the science involved and the research process involved. What is the lay summary, goals and timeline?” |
| **Engagement as challenge** |  | **COM:**  **“**Engagement still a challenge.”    **“**Sometimes may not be available; especially hard to engage youth during the work day.” |
| **Group homogeneity** |  | **COMM:**    **“**Members of the CEC are mostly parents. It would be great to diversify to have a more broad range of perspectives (more fathers, young people, Indigenous community-partners, etc).”    **RES:**    **“**Naturally some voices are louder than others, but we made adjustments in the last year to ensure that each individual voice felt heard. The demographics of our parents are fairly similar but their perspectives are still incredibly valuable”    “I'm always concerned that patient-partners may not be representative of minorities. However, as so well stated by one expert patient-partner, the biostatistic consultant is not representative of all biostatisticians, but does have an expertise in the domain. So I have come to feel more confortable that patient-partners are experts of their domain and will ensure that the research we conduct reach all patients, including minorities.”    **“**We also want to have a diversity of perspectives in each task, such as including at least a youth and parent partner.” |
| **Keep up with engagement over long periods of time** |  | **RES:**  **“**We had a lot of really good patient engagement at study start but it has been harder to engage our entire group as the study has gone on.”    **“**I would say that having the initial meeting with parents was sometimes difficult in terms of knowing when they were on the NICU to present the project and the follow-up is sometimes challenging to do the EPDS once they have been discharged.” |
| **Lack of clarity re roles** |  | **PAG:**    **“**I was put on the project by a neonatologist in our Health Centre. It would have been helpful to have some context right away and a terms of reference for the Child-Bright initiatives. The EPIQ projects are amazing!”    **“**Patient-partners don't need any supports different from those other members need. What the whole committee needs is greater clarity about the role of the NSC and their roles within the NSC”    **“**The academics/investigators are very focused on their individually funded research projects (and likely other research/admin/teaching commitments at their home institution) - this doesn't leave much energy/interest in new research initiatives that might emerge from our experiences as a Network. Also, I can't figure out the mandate of the research committee - I'm not sure we've done a great job at identifying the key issues across projects that we could then tackle as a Network - some of them feel out of our control (e.g. contracts/agreements, ethics), but maybe there are things that we could be doing to support one another across projects differently? I'm not sure...I'm also to blame as I'm now realizing that I haven't bothered to ask anyone what the mandate of the committee is and how can I best contribute!” |
| **Lack of F2F meetings** |  | **PAG:**  **“**We don't get to meet in person often enough.” |
| **Lack of meetings or PP presence in meetings** |  | **COMM:**  **“**The Committee meets so infrequently that it is hard to respond to this question.”    “Again, most of the research committee meetings I've been able to attend do not seem to have a patient partner in attendance (not to say there isn't one on the committee), so it's difficult to assess.”    “There is only one patient-partner on the committee, so in some ways that limits the number of perspectives that they bring. However, because the patient-partner is so experienced, they are able to speak for more than just themselves.” |
| **Lack of PI engagement** |  | **PAG:**  **“**The study leadership not as engaged. Come to calls late, often give the responsibilities to their colleagues and/or often seem distracted or leave early.” |
| **Linguistic, social and cultural differences** |  | **RES:**  **“**Linguistic, social, and cultural differences limit or may prohibit many study participants from full engagement.” |
| **Managing PP expectations vs feasibility in incorporating feedback** |  | **RES:**  **“**Managing PFAC expectations agains the feasibility of incorporating their feedback to the project, the RCT and the IT development. Being assertive and vigilant on the project baselines (scope, time and budget).”    **“**A challenge of partnering with the PFAC is that we want to ensure that we take the time to incorporate their perspectives into each stage of the project.” |
| **Newsletters hard to navigate** |  | **PAG:**  **“**I find the information hard to navigate in the newsletters.” |
| **Pandemic related issues** |  | **RES:**  **“**Note: Current challenges related to COVID-19 situation might make it difficult for parent-advisors to be engaged in Network activities (e.g. the current survey) and findings time/efforts/motivation to participate. We need to be sensitive to this situation and have a more individualized approach (instead of group automatic emails) when we try to reach them” |
| **PE is time consuming** |  | **RES:**  **“**At times it takes more time.” |
| **PIs too focused on individual research projects** |  | **COMM:**    **“**The academics/investigators are very focused on their individually funded research projects (and likely other research/admin/teaching commitments at their home institution) - this doesn't leave much energy/interest in new research initiatives that might emerge from our experiences as a Network. Also, I can't figure out the mandate of the research committee - I'm not sure we've done a great job at identifying the key issues across projects that we could then tackle as a Network - some of them feel out of our control (e.g. contracts/agreements, ethics), but maybe there are things that we could be doing to support one another across projects differently? I'm not sure...I'm also to blame as I'm now realizing that I haven't bothered to ask anyone what the mandate of the committee is and how can I best contribute!” |
| **Planning ahead for resources needed to do engagement** |  | **RES:**    **“**The biggest challenge projects face generally is not planning ahead for the extra resources needed to do engagement well. For example, - not having a dedicated central staff person to coordinate activities and connect with PFAC members outside of meetings. - not having budget lines for patient compensation, supports for training, registration, travel to attend conferences - not being realistic when planning their timelines (doing engagement well takes more time) CHILD-BRIGHT as a network, and this project specifically both have made good strides in these areas but it took some time to get there.” |
| **Possibly overwhelming PP with tasks** |  | **RES:**  **“**Sometimes I feel that they are getting too many links/surveys at once and might feel overwhelmed with the tasks they need to do. I propose to have a central database displaying ongoing and future tasks and their progress, to make sure we do not overwhelm our members” |
| **Project related technical difficulties** |  | **RES:**    **“**We experienced technical issues with the App during which not much was happening in the research project, during this time we had not been able to update our patient partners as much as before (we used to send bi monthly infographics with projects updates, which we will restore.” |
| **Scheduling and organizing meetings that suit everyone’s availabilities** |  | **RES:**    **“**Scheduling and finding optimal times to meet is always a challenge. Families are busy. We have made sure to provide a range of options and locations to facilitate finding the most convenient times.”    **“**Scheduling team meeting at times that work for everyone, particularly trying to get everyone in the same room. We have begun to rotate lunch and after work meetings to better accommodate members' schedules - this way if the timing isn't great for one meeting for some members, it will be better during the next meeting.”    **“**It is sometimes difficult to coordinate a committee consisting of researchers, physicians, young adults/youth with CP, and caregivers; everyone has unique circumstances and are not often available at the same time. We would love to have meetings even more regularly but need to take into account the burden placed on members.” |
| **Struggle to establish formal relationships with PP** |  | **RES:**    **“**Trial is on track overall (first question) and we have active informal engagement with many families but have struggled to establish more formal relationships despite numerous invitations (aside from above committee members).” |
| **Time zones and different locations** |  | **PAG:**    **“**Time zones! Participating from Alberta can be hard logistically.”    **RES:**    **“**I think we can do a better job in setting expectations and meeting our objectives. While we make a good attempt, in all honesty I think we have missed out on being true to co-creation as there is a PFAC and a core-research team that operates in parallel, rather than integrated. Moving forward my plans is for the RCT to have a more integrated approach. The benefits are obvious, I think the materials have benefited a lot from the PFAC influence. The challenges are to keep it all together in such a big and complex project, with a research team that has not worked together before, is situated in different locations. “ |
| **Transition in project phases and its impacts on engagement** |  | **RES:**    **“**The transition from development to data collection, where the type of engagement changed significantly. A recommendation would be to plan for how the roles of our committee changes over the course of project, to better communicate with our parents and transition between phases.”    **“**The only 'challenge' would be the evolving levels of engagement between the different phases within one project. A recommendation would be to map out a timeline of engagement over the course of the project to better set expectations of the parent advisors and to better transition methods of engagement between phases” |

Legend: Committee member (COMM); Member of the parent-group advisory (PAG); Researcher (RES).

**Facilitators to patient engagement**: **Most salient utterances**

| **Theme** | **Sub-theme** | **Examples of participants’ quotes** |
| --- | --- | --- |
| **Communication strategies** | **Adequate preparation for meetings** | **COMM: “**Members are regularly asked to add items to the agenda. We also expanded the length of the meetings in large part to make it easier for all to contribute and not feel squeezed by time. I think I (as chair) should contact members more often between meetings to check in about how they think things are going.”    “Over time, a certain rapport or camaraderie has been established throughout the membership. Each meeting usually had members share unique and valuable perspectives. Where applicable, one-on-one meetings are arranged with patient-partners to further discuss items or plan a course of action.”    **RES:**    “Since the inception of the PFAC, we have had monthly meetings with the full PFAC team to provide updates and have discussions about the project. The PFAC has also been supported in their participation with our individual check-in meetings to ask them about their expectations, goals, and responsibilities of their involvement in this project. These check-in meetings were helpful in our plans moving forward, in which we plan to have subgroup meetings for tasks that PFAC members are interested in and 3-4 full PFAC meetings throughout the year. PFAC members are also welcome to email the research coordinators of the project outside of the monthly meetings to ask for updates. We also share meeting minutes, so that PFAC members can continue to be updated and/or ask questions outside of the monthly meetings.” |
|  | **Consistent and clear communications** | **PAG:**  **“**More consistent and clear information shared between sites.” |
|  | **F2F meetings** | **PAG:**  **“**The face to face meetings in Montreal at the start of the project were pivotal in creating trust and confidence for sharing on the team.” |
|  | **Pauses in meetings for questions, encouragement for questions** | **COMM:**  **“**During the meeting, pauses are taken for questions to be asked, which allow members to have a better understanding of the topics that are being discussed. Questions are often encouraged and well received.“ |
|  | **Smaller group meetings for better collaborations** | **PAG:**  **“**Keep having team meetings in smaller groups so everyone can collaborate better.” |
| **Improved supports to PP** |  | **RES:**  **“**ChildBright has greatly improved their support of patient partners since the beginning of the study.”    “Support are mostly adequate for patient-partners to fully participate in the IMAGINE project. When they are unable to participate, it is usually for care-giving reasons - their child is too ill to be left.” |
| **Methods of engagement based on feedback** |  | **RES:**    **“**As the project moved from development in to data collection, we adjusted and individualized our methods of engagement based on feedback from our parents. So while we do our best to provide support and enough information, we are always making adjustments to do better”    “The methods in which we support and engage our parent advisors are iterative, and have gone through recent changes to accommodate the changing levels of needs/engagement. Sometimes our parents do not have enough information to participate BUT we have a strong relationship whereby they can indicate their needs” |
| **Opinions being welcomed, acknowledged, heard, and respected** |  | **PAG:**  **“**I'm able to say what I want to say and I feel everyone's views are taken into consideration. Criticism is professional and constructive.”    **COMM:**  **“**Not a whiff of tokenism. My perspective and experience as a patient-partner are valued by all the other members.”    **RES:**  **“**I sincerely hope that PFAC members feel that their contributions and insights / views are valued and that there is a trustful relationship / safe environment to share their views and perspectives. I welcome any feedback an opportunity to learn and grow together.” |
| **PIs being responsive to requests from PP** |  | **RES:**  **“**The have been responsive to requests from partners.” |
| **PP active in KT** |  | **RES:**  **“**Our patient-partners have beeen key players in the knowledge transfer component (presentations to researchers and clinicians that were very impactful).” |
| **PP feeling supported** |  | **COMM:**    **“**I feel supported and welcomed to share my perspectives as a trainee member of the Training Committee. I am slowly learning about the different initiatives that the Training Committee is involved in, and they have been opened to asking for feedback from everyone.”    **“**As a Trainee, I have been able to share my perspectives about insights of how trainees can be supported to learn and engage in patient-oriented research. I have had positive experiences with being a part of the Training Committee, because my perspectives are taken into account. For example, I have shared about different learning opportunities that can help to have more trainees involved such as with virtual post presentations.” |
| **PP involved early in project** |  | **RES:**  **“**Patient partners were involved from the outset of the project and are aware of the value their experience provides. They are an integral part of project success.” |
| **Qualities of PP** |  | **PAG:**  **“**Open, engaged, thoughtful participants”    **RES:**  “I think our project has invested in and continues to do in establishing a positive relationship between patient/family members and researchers. I am impressed by the commitment by the patients (youth with BBDD) and parents for so long. The project is now in its 5th year and we are about to start the clinical trial.”    “The patient partners in the IMAGINE project are independent thinkers who do not appear to hesitate to contact our staff regarding any topics.” |
| **Researchers and PP strong partnership and understanding, mutual goals** |  | **RES:**    **“**feedback from our personal check-in calls with PFAC members: Above all they see and appreciate that the research team is trying to make this experience valuable and meaningful - making the effort! We are learning together and not always doing everything 'right' but it's clear to them that we value the partnership, are open to considering how we can take up everyone's input to enrich the project and also to continue to build the relationship and collective goals beyond this project's aims.” |

Legend: Committee member (COMM); Member of the parent-group advisory (PAG); Researcher (RES).

**Strategies to improve patient engagement: Most salient utterances**

| **Theme** | **Sub-theme** | **Examples of participants’ quotes** |
| --- | --- | --- |
| **Actions taken to ensure supports and mitigate barriers** |  | **RES:**  **“**When patient-partners have voiced their perspectives of barriers and supports, they are aware of actions subsequently taken to ensure those supports continue and to mitigate barriers as able within the study process.” |
| **Allow more flexibility in PP engagements** |  | **RES:**  **“**Parents are not always available, as they have a lot on their plate and may at times be experiencing greater stresses. It is important to have a large enough parent advisory team, so that a subset can disconnect when necessary and not feel the pressure of responding and interacting with the team at all times. It is key to allow the flexibility for parents to participate when possible.” |
| **Appreciation initiatives** |  | **RES:**    **“**Regular engagement, including them throughout the process and not only when challenges come up that we need help with. Regular team building support at each site that is consistent across sites. For example monthly coffee meetings with the whole team (coach, PI, RAs), sending parent-advisors thank you/birthday cards, brainstorming other ways to make them feel appreciated. This may be happening at a nation level, but I think more could be done to build team morale and support at local sites.” |
| **Appropriate renumeration and other incentives** |  | **COMM:**    **“**Appropriate remuneration. The more that we create processes where they share in true co-production, the more they should be paid in a way that moves beyond honoraria.”    **“**Although very dedicated, there is a hesitation to overburden patient-partners with specific tasks or to make them responsible for particular outputs. This could likely be overcome with added funding support so that the partner does not just volunteer their time.”    **RES:**    **“**By definition, patient partners are caring for children with varying medical needs. This can make life very busy for them and requires flexible scheduling, and creative problem solving. It also means that we need to continue to budget for ongoing remuneration for their time and travel especially considering the sacrifices they are making to provide invaluable input to our research.” |
| **Central database of to dos for PP to make sure no one is overwhelmed with assignments** |  | **RES:**  **“**Sometimes I feel that they are getting too many links/surveys at once and might feel overwhelmed with the tasks they need to do. I propose to have a central database displaying ongoing and future tasks and their progress, to make sure we do not overwhelm our members” |
| **Clarity on goals, out puts and contributions** |  | **PAG:**    **“**i definitely feel like more education on the importance of family participation in rec activities would help increase capacity for what you are intending to do”    **COMM:**  **“**I"m not sure how patient-partners could be better engaged in the main research committee - but if the research committee had a clear mandate and perhaps a set of priority initiatives that we were to tackle as a committee, then I think having patient partners engaged would be highly valuable (esp given the experience we have had with the data reuse taskforce). We've started another initiative with SPOR CDP / DASH - maybe this could be shaped as more formal taskforce as well with patient partner engagement?”    **RES:**    **“**I think we can do a better job in setting expectations and meeting our objectives. While we make a good attempt, in all honesty I think we have missed out on being true to co-creation as there is a PFAC and a core-research team that operates in parallel, rather than integrated. Moving forward my plans is for the RCT to have a more integrated approach. The benefits are obvious, I think the materials have benefited a lot from the PFAC influence. The challenges are to keep it all together in such a big and complex project, with a research team that has not worked together before, is situated in different locations.  Although Patient-Oriented Research could be a times challenging, as it impacts on the project baselines and had impacted on the READYorNot App development, it has been very useful and appreciated. The point has been to be able to manage their expectations against the feasibility of incorporating their feedback to the project, the RCT and the IT development.” |
| **Clearer goals re meetings** |  | **COMM:**    **“**would recommend being more clear about the purpose of specific meetings and what input is sought from patient partners for each specific meeting.”    “I would recommend that goals for specific meetings are more clearly laid out, so that patient partners know for which purpose their input is being sought.” |
| **Crosslinkages between committees** |  | **RES:**    **“**A challenge of partnering with the PFAC is that we want to ensure that we take the time to incorporate their perspectives into each stage of the project. We also want to have a diversity of perspectives in each task, such as including at least a youth and parent partner. To maximize the engagement of PFAC and family partners in the CHILD-BRIGHT network, I wonder whether there could be opportunities for cross-linkages, such as being able to connect with other committees such as the Citizen Engagement Committee and/or Knowledge Translation Committee.” |
| **Ensure balance between PP voices** |  | **RES:**    **“**Naturally some voices are louder than others, but we made adjustments in the last year to ensure that each individual voice felt heard. The demographics of our parents are fairly similar but their perspectives are still incredibly valuable”    **“**There are always stronger voices than others, and this was something we recognized and adapted too. Some parents contribute better 1:1 whereas others thrive in group settings, we accommodate all types of participants. Our parents come from similar socio-economic backgrounds, and have recognized that themselves, however, that does not diminish their individual, invaluable contributions.” |
| **Finding balance between partners and youth partners’ voices** |  | **COMM:**    **“**One challenge has been insuring that the parent-partners' views and voices don't dominate, especially in relation to those of the two youth members. We also need to include more non-parent and non-youth members, in part, for all the perspectives to nourish and challenge the others.” |
| **Inperson meetings** |  | **RES:**    **“**More opportunities/funding to meet in person” |
| **Keep up with promises to give feedback** |  | **COMM:**    **“**We still have some promises to keep with respect to some of the initiatives that they have brought forward, particularly, ensuring that families/participants receive some feedback at the end of the project. This doesn't have to necessarily be the papers that are published as a result of the participants' enrolment, could just be a general statement of how the findings have influenced the field/lab, where possible”    **“**It is important to continue to include patient-partners in the Training Committee with ongoing communication, such as continuing to welcome feedback and input through emails if members are not able to attend the meetings. One way to improve the engagement of patient-partners is to share how their feedback was incorporated in different tasks.” |
| **Making sure to act on all recommendations** |  | **COMM:**    **“**We've acted on most of the CEC's recommendations / suggestions, but we (I especially) should do more to ensure we act on all (e.g. the toolkit that keeps coming up but that we haven't really acted upon).”    **“**ensuring that we consider the patient voice to inform all decisions and strategic directions of the network” |
| **Managing expectations** |  | **COMM:**    **“**Managing expectations things happen slow in the medical world” |
| **PP on REB reviews** |  | **PAG:**    **“**More involvement is needed to include patient partners in REB reviews and considerations” |
| **Recruit more diverse members** |  | **PAG:**  **“**More parents on each team would be great.”    **COMM:**  **“**it would be great to have a youth; however it is difficult to have that type of regular commitment during the workday.”    **RES:**  **“**It would be ideal if we had a greater diversity of our patient partners (e.g., diversity with respect to race, gender, SES) to have a broader perspective. Our patients in the health care system are diverse, and this is not reflected in our committee.” |
| **Redirect funding to better support PPs in different initiatives** |  | **RES:**    **“**For challenges, see previous comments on inclusion of those "seldom heard." If we had more time = more funding - I feel we could take the time to work with various communities to find how to better support those who are not active patient partners. As an ex-public health nurse, I know the value of "going to where the family is" and that includes home visits. I would love to see a position funded for our project specifically for this. I would recommend spending less on Child Bright central - less for conferences, workshops etc, and divert those funds specifically for an outreach position for projects like ours where there is an identified need.” |
| **Strengthen communication channels** |  | **COMM:**    **“**It is important to continue to include patient-partners in the Training Committee with ongoing communication, such as continuing to welcome feedback and input through emails if members are not able to attend the meetings. One way to improve the engagement of patient-partners is to share how their feedback was incorporated in different tasks.”    **RES:**  “There are always stronger voices than others, and this was something we recognized and adapted too. Some parents contribute better 1:1 whereas others thrive in group settings, we accommodate all types of participants. Our parents come from similar socio-economic backgrounds, and have recognized that themselves, however, that does not diminish their individual, invaluable contributions.”    “The transition from development to data collection, where the type of engagement changed significantly. A recommendation would be to plan for how the roles of our committee changes over the course of project, to better communicate with our parents and transition between phases.” |
| **Team building exercise** |  | **RES:**    **“**Regular engagement, including them throughout the process and not only when challenges come up that we need help with. Regular team building support at each site that is consistent across sites. For example monthly coffee meetings with the whole team (coach, PI, RAs), sending parent-advisors thank you/birthday cards, brainstorming other ways to make them feel appreciated. This may be happening at a nation level, but I think more could be done to build team morale and support at local sites.” |

Legend: Committee member (COMM); Member of the parent-group advisory (PAG); Researcher (RES).
